# Probing the salt dependence of the torsional stiffness of DNA by multiplexed magnetic torque tweezers

**DOI:** 10.1093/nar/gkx280

**Published:** 2017-04-29

**Authors:** Franziska Kriegel, Niklas Ermann, Ruaridh Forbes, David Dulin, Nynke H. Dekker, Jan Lipfert

**Affiliations:** 1 Department of Physics, Nanosystems Initiative Munich, and Center for Nanoscience, LMU Munich, Amalienstrasse 54, 80799 Munich, Germany; 2 Department of Bionanoscience, Kavli Institute of Nanoscience, Delft University of Technology, Van der Maasweg 9, 2629 HZ Delft, The Netherlands; 3 Junior Research Group 2, Interdisciplinary Center for Clinical Research, Friedrich-Alexander-University Erlangen-Nürnberg (FAU), Hartmannstrasse 14, 91052 Erlangen, Germany

## Abstract

The mechanical properties of DNA fundamentally constrain and enable the storage and transmission of genetic information and its use in DNA nanotechnology. Many properties of DNA depend on the ionic environment due to its highly charged backbone. In particular, both theoretical analyses and direct single-molecule experiments have shown its bending stiffness to depend on salt concentration. In contrast, the salt-dependence of the twist stiffness of DNA is much less explored. Here, we employ optimized multiplexed magnetic torque tweezers to study the torsional stiffness of DNA under varying salt conditions as a function of stretching force. At low forces (<3 pN), the effective torsional stiffness is ∼10% smaller for high salt conditions (500 mM NaCl or 10 mM MgCl_2_) compared to lower salt concentrations (20 mM NaCl and 100 mM NaCl). These differences, however, can be accounted for by taking into account the known salt dependence of the bending stiffness. In addition, the measured high-force (6.5 pN) torsional stiffness values of *C* = 103 ± 4 nm are identical, within experimental errors, for all tested salt concentration, suggesting that the intrinsic torsional stiffness of DNA does not depend on salt.

## INTRODUCTION

DNA is the carrier of genetic information in all cellular life. In addition to its sequence, its polymeric and mechanical properties are critical for its biological function. For example, in our cells ∼2 m of DNA are packed into ∼10 μm-sized nucleus (1), involving a multitude of deformations and DNA–protein interactions. *In vivo* DNA is negatively supercoiled and both supercoiling and DNA packing play important roles in gene regulation (2–7). In addition to its biological role, DNA is emerging as a building block for artificially created 3D nanostructures (8). A detailed and quantitative description of DNA mechanical properties is a prerequisite to understanding its biological roles and for its optimal use as a construction material at the nanoscale.

At length scales significantly longer than 1 bp, DNA is commonly modeled as an isotropic elastic rod, with a bending persistence length *A*, stretch or Young's modulus *B*, torsional stiffness *C* and twist-stretch coupling *D* (9,10). All four elastic constants have been determined for DNA *in vitro* in a series of ingenious single-molecule manipulation measurements (11–20). However, their values, in general, can depend on solution conditions. In particular, salt concentration can strongly modulate DNA properties, as DNA is highly negatively charged (21). In a classic argument, Odijk (22) and independently Skolnick and Fixman (23) derived how electrostatic repulsion increases DNA bending stiffness, *A*, and how the bending persistence length can be separated into a salt-independent and a salt-dependent contribution. Subsequently, single-molecule stretching experiments have determined the salt dependence of *A* for DNA (24,25) and recently also for RNA (26,27) and indeed found a reduction of *A* by ∼20% going from low (<20 mM monovalent salt) to relatively high (>300 mM monovalent or ∼10 mM divalent salt) ionic strength. However, the exact nature and molecular details of this dependence remain an area of active research (28–38).

The essence of the Odijk and Skolnick-Fixman argument is that bending a charged rod will bring charges into closer proximity, which will cost more energy if there are fewer screening counterions and, therefore, the stiffness will be increased at low salt. In contrast, for a homogenously charged rod, twisting the rod does not alter the distance between charges, which would suggest the twist-stiffness not to depend on salt. For real molecules the charges are localized, though; in DNA, the phosphate groups carry the negative charges under physiological conditions and it is not *a priori* clear whether the torsional stiffness would depend on salt.

Several properties of DNA under torsional constraint, in particular aspects of DNA supercoiling, are known to strongly depend on salt concentration (39–42). Under sufficient torsional stress, DNA undergoes a buckling transition and forms plectonemic supercoils. Due to electrostatic repulsion, the diameter of plectonemic supercoils increases with decreasing salt concentrations (43–46). In addition, the critical linking number for buckling (19,47), the number of plectonemes (48,49), as well as their dynamics (42) have all been found to depend on salt concentration. However, plectonemic supercoils involve strong bending and bring DNA segments into close proximity and these observations, therefore, do not directly probe the torsional stiffness in isolation. Neukirch suggested from theoretical analysis of magnetic tweezers (MT) measurements on supercoiled DNA that the torsional stiffness of DNA decreases almost 2-fold upon addition of millimolar concentrations of Mg^2+^ ions (43). Similarly, direct torque measurements using a rotor bead assay suggested a small decrease of the torsional stiffness with increasing ionic strength (∼10% in going from buffer only to ∼150 mM monovalent salt and no additional decrease, within experimental error, upon further addition of ∼1.8 M monovalent salt; at 10 pN stretching force) (50). In contrast, Delrow *et al*. found an increase in torsional stiffness in fluorescence polarisation anisotropy (FPA) measurements by ∼20% upon addition of 5 mM Mg^2+^ (51). Finally, indirect torque measurements by thermodynamic integration (19) and Monte Carlo modeling (45) of MT measurements on supercoiled DNA concluded the torque at and past the buckling transition to depend on salt, but suggested little dependence of the torsional stiffness on ionic strength. In summary, the dependence of the torsional stiffness of DNA on salt concentration and valence is currently debated, in particular due to the lack of systematic and direct measurements.

**Figure 1.**
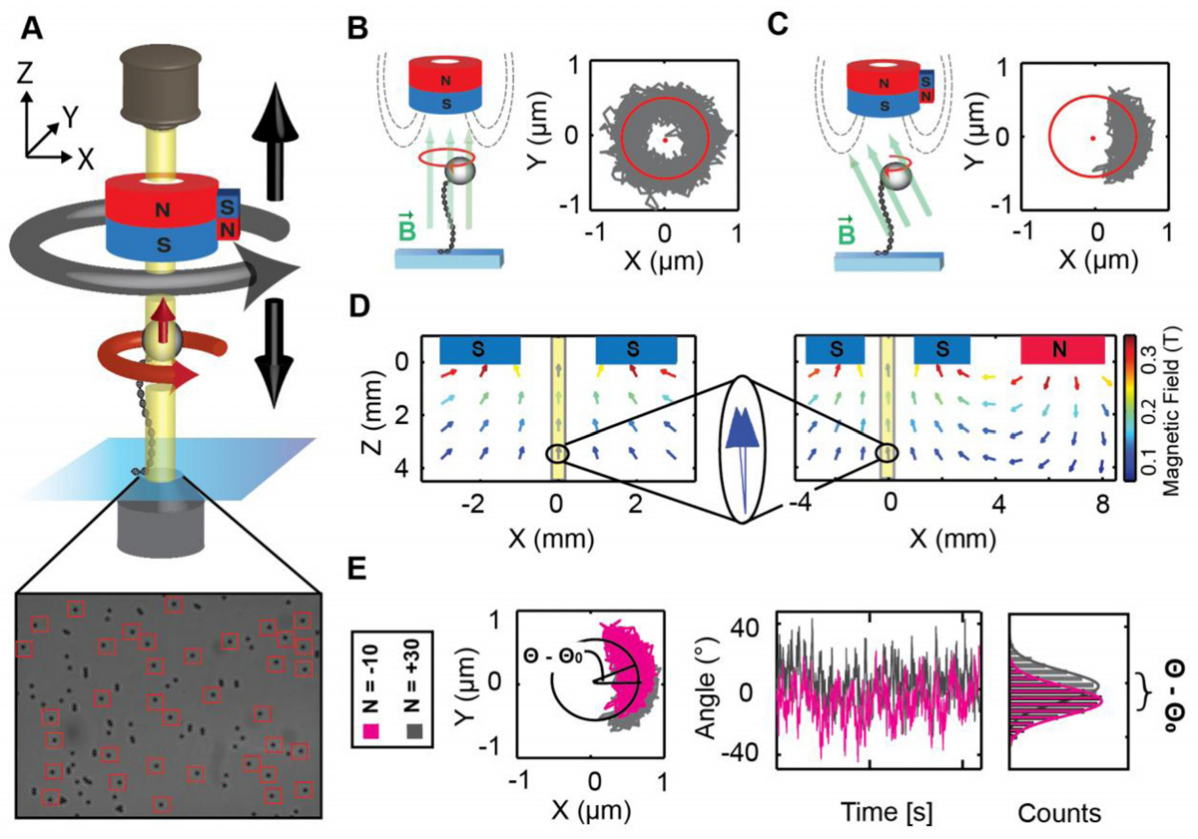
Principle of magnetic torque tweezers. (**A**) A schematic of mMTT. The molecules are tethered between magnetic beads and a glass coverslip. Permanent magnets, placed above the flow cell, exert magnetic fields, therefore enabling the application of forces and torques to the tethered molecules. The beads’ positions are monitored by video tracking using an inverted microscope and monochromatic illumination. mMTT allow tracking of multiple beads in parallel (bottom left, red frames indicate beads marked for tracking), here 36 M270. (**B**) In FOMT, the bead's anisotropy axis aligns with the vertical magnetic field, exerted by the cylindrical magnets. The bead's motion is (torsionally) unconstrained and traces out a doughnut-like shape in the (*X,Y*)-plane. (**C**) An additional side magnet in the (m)MTT slightly tilts the magnetic field. This leads to (weak) angular trapping of the bead. Its motion in the (*X,Y*)-plan is confined to an arc-like shape. (**D**) Simulations of the magnetic fields for FOMT (left) and MTT (right) show that the difference in the magnetic field is small (large, blue arrows). The yellow shaded areas in the magnetic field simulations represent the width of the field of view. (**E**) The tracked (*X,Y*)-position of the bead is transferred to polar coordinates. Upon turning the magnets *N* times, the molecule exerts a restoring torque, causing a shift in the equilibrium position (*Θ* − *Θ*_0_) of the rotation angle. Data shown are for *F* = 3.5 pN, going from *N* = −10 turns to *N* = +30 turns.

Here, we demonstrate multiplexed magnetic torque tweezers (mMTT) that enable parallelized single-molecule torque measurements. Using this experimental approach, we determine the torsional stiffness of DNA as a function of applied stretching force and salt concentration. Our mMTT instrument is an extension of conventional MT that have proven to be a powerful tool to investigate the torsional properties of nucleic acids (10,52,53). In MT and mMTT, molecules of interest are tethered between a flow cell surface and magnetic particles (Figure 1). Bead positions are tracked by video microscopy, which enables tracking of many beads in parallel (54–56). Stretching forces are exerted by applying external magnetic fields that pull the magnetic particles upward. The forces can be calculated from the magnetic field gradients by taking into account the induced magnetization on the beads (57) and experimentally determined from analysis of the transverse fluctuations (52,58–61). Conventional MT can apply torque by rotating the external magnetic field, which in turn rotates the beads, however, they do not measure torque. Torque measurements on individual DNA molecules in the MT have been achieved using an indirect approach by thermodynamic integration (19) and directly by tracking the rotation angle, either of a rotor bead (15,17,50,62) or of the magnetic bead or assembly (18,20,63–65). Our mMTT implementation employs an optimized angle tracking protocol and magnet geometry to extend the multiplexing capabilities inherent in MT measurement to the torque dimension.

## MATERIALS AND METHODS

### Magnetic tweezers instrument

Measurements were performed with a home-built MT setup. In brief, a light emitting diode (Osram Oslon SSL, red, 165lm) is used to illuminate the sample, monochromatically from above. An oil immersion objective (60× Plan Fluorite with correction collar, NA 0.9 or 40× Plan Fluorite, NA 0.75, Olympus) is placed on a piezo (Pifoc, P-726.1CD and controller, E-753.1CD, Physik Instrumente) stage underneath the flow cell holder. The flow cell is imaged with a mirror (20D20ER.1, Newport) and tube lens (G322304000, Newport) onto the chip of a camera (Falcon PT-41-4M60, Dalsa). Magnets (see below) are placed on a motorized arm, using a translational motor (C-863.11-Mercury controller and M-126.PD2 motor, Physik Instrumente) and a rotational motor (C-863.11-Mercury controller and C-150.PD motor, Physik Instrumente) to control the magnets’ rotation and position in *Z*. The flow cell outlet is connected to a pump (ISM832C, Ismatec) for fluid handling (66). The setup is controlled using a computer (DELL Precision T3600) equipped with a framegrabber (PCIe-1433, National Instruments) and using software written in LabVIEW (National Instruments) described by Cnossen *et al.* (56).

### DNA construct and beads for MT measurements

Measurements used a 7.9-kbp DNA construct as previously described (20). Specific and torsionally constrained coupling of the DNA to magnetic beads (streptavidin-coated 1.0 μm diameter MyOne or 2.8 μm diameter M270 beads; Life Technologies) and the flow cell surface was achieved through ligation with ∼600-bp polymerase chain reaction-generated DNA fragments, comprising multiple biotin- and digoxigenin-modified dUTP moieties (Jena Bioscience), respectively. The DNA construct was first coupled to the streptavidin-coated beads by incubating 5 ng of the DNA construct with 2 μl of MyOne (or 5 μl M270) beads in a final volume of 20 μl of phosphate buffered saline (PBS; Sigma-Aldrich) for 12 min (5 min for M270 beads). The DNA-bead solution was subsequently diluted into 100 μl PBS and introduced into the flow cell.

### Preparation of the flow cell

Flow cells were constructed by assembly of two glass coverslips (24 × 60 mm, Carl Roth) separated by a single parafilm (PARAFILM M, H666.1, Carl Roth) layer that formed a flow channel used as the measurement chamber. The bottom coverslip was first modified using (3-Glycidoxypropyl)trimethoxysilane (abcr GmbH), subsequently reacted for one hour with anti-digoxygenin (100 μg/ml in 1× PBS; Roche) and then passivated using a commercial passivation mix (BlockAid™ Blocking Solution, Thermoscientific) for 1 h. After excessive flushing of the flow cell using PBS buffer, the DNA-bead solution is introduced and allowed to bind for ten minutes (MyOne beads) or 50 s (M270 beads). Unbound beads are removed from the flow cell by flushing with ∼500 μl PBS.

### Bead selection

**Figure 2.**
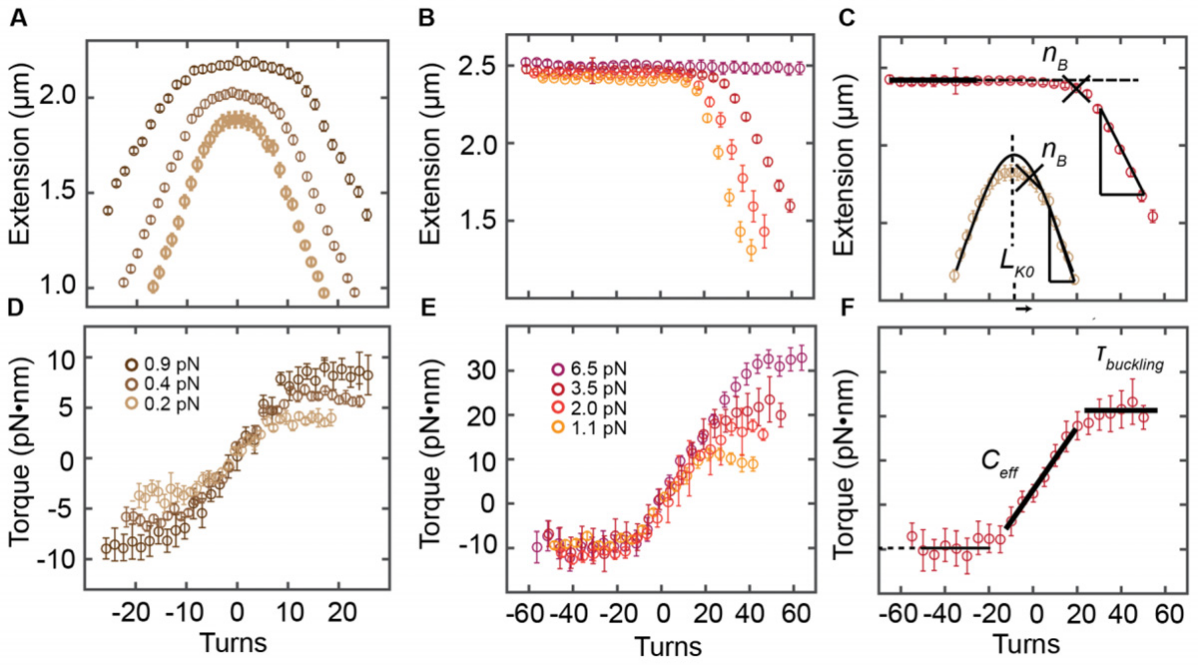
Averaged extension-rotation and torque-rotation measurements of dsDNA at varying forces. Measurements shown were recorded in TE buffer at pH 7.4 with 100 mM NaCl. (**A** and **D**) At low forces (<1 pN; indicated in the legend in panel D) the extension versus turn plot is symmetric about zero turns. When applying a number of turns to the DNA molecule (positive and negative), its extension stays constant, while the molecular torque increases linearly. Beyond the buckling point (post-buckling) the extension decreases linearly with each additional turn, while the molecular torque stays constant. (**B** and **E**) For higher forces (>1 pN; indicated in the legend in panel E), no plectoneme formation occurs upon underwinding the molecules. Instead, torque induced melting takes place at −10 pN·nm. For forces >6 pN, no supercoiling occurs at all. Instead a transition from B- to P-DNA occurs upon overwinding at 35 pN·nm. (**C**) Determination of the buckling points (*n_B_*; black triangles), the post-buckling slopes (black lines) and *Lk_0_* (only for symmetric extension-rotation curves). (**F**) A line fit to the linear regime in the torque-rotation data (black line) is used to determine the torsional stiffness (*C_eff_*). The mean of the constant plateau for positive turns (beyond buckling) is used to compute the buckling torque (*τ_buck_*; black horizontal line).

To verify that selected beads are bound to a single, torsionally constrained DNA tether, several tests were performed using conventional MT that employed a pair of cubic permanent magnets (5 × 5 × 5 mm^3^; W-05-N50-G, Supermagnete, Switzerland), oriented in a vertical configuration (57) with a spacing of 1 mm. First, the external magnets are moved vertically to exert alternating forces of ∼5 pN (to fully stretch the molecule) and <0.01 pN (close to zero force) in order to test the contour length of the tethers. The measured change in extension is expected to be approximately the contour length of the DNA double-strand. Next, magnets are rotated clockwise by 20 turns at high (5 pN) tension to test for multiple tethers: At forces >1 pN, DNA does not form plectonemic supercoils upon unwinding (corresponding to counterclockwise rotation), but melts instead (67) and no decrease in the extension is expected for single DNA tethers (Figure 2B). In contrast, if a bead is attached via two or more double-stranded DNA molecules, the molecules will form braids if the bead is rotated, causing a decrease in the molecule's extension, independent of stretching force. To select for beads that are coilable (i.e. with a fully double-stranded backbone and attached via multiple attachment points at both ends) magnets are rotated by 20 turns in both directions at 0.6 pN to check for DNA supercoiling (Figure 2A), which is seen in a decrease in the detected extension. Finally, the flow cell was flushed with ∼500 μl of the appropriate measurement buffer, see Table 1.

**Table 1.** Buffer conditions and salt concentrations

	Tris	EDTA	NaCl	MgCl_2_
**Salt 1**	10 mM	1 mM	20 mM	0
**Salt 2**	10 mM	1 mM	100 mM	0
**Salt 3**	10 mM	1 mM	500 mM	0
**Salt 4**	10 mM	1 mM	100 mM	10 mM

Measurements were performed in TE-buffer at pH 7.4. Ionic strength was varied by the addition of NaCl (salt 1, 2 and 3) or a combination of NaCl and MgCl_2_ (salt 4).

### Magnetic torque tweezers configuration and measurement protocol

Conventional MT do not allow direct measurements of torque (20); for single-molecule torque measurements the cubical magnets are, therefore, replaced by a set of cylindrical magnets (Figure 1A) that are assembled as a stack of three ring magnets with an outer diameter of 6 mm, a 2 mm diameter aperture in the center and a height of 2 mm per magnet (R-06-02-02-G, Supermagnete, Switzerland). A cylindrical magnet with 3 mm diameter and 6 mm height (S-03-06-N, Supermagnete, Switzerland) is used as a ‘side magnet’. This magnet configuration exerts an upward pulling force (20,60) while providing a weak rotational trap suitable for torque measurements. Details and optimization of the magnet configurations are further discussed below. Between rotating the magnets, we measured the angle fluctuations at a fixed number of applied turns for 100–200 s for MyOne and M270 beads. The characteristic time scales of rotational fluctuations are ∼0.1 and ∼0.5 s for MyOne and M270 beads, respectively, in MTT representative for our conditions (20,64,65). Our measurement intervals, therefore, correspond to >100 times the characteristic time scale of angular fluctuations. We note that our camera integration time of ∼17 ms (corresponding to the frame rate of 60 Hz) is significantly shorter than the characteristic time scale of rotational fluctuations, making blurring and aliasing effects negligible (59,68). The rotation speed of the magnets for under- and overwinding of the DNA tethers by integer number of turns during torque measurements was set to 0.1 Hz.

### Data averaging of multiple single-molecule measurements

**Figure 3.**
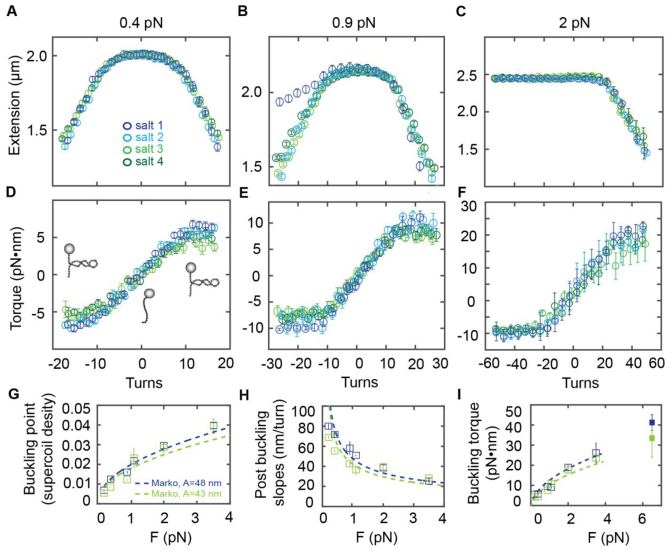
Averaged extension-rotation and torque-rotation responses of dsDNA for different salt conditions. Averaged extension versus turns data for four different buffers and corresponding averaged torque versus turns data at 0.4 pN (**A** and **D**), 0.9 pN (**B** and **E**) and 2 pN (**C** and **F**). Blue colors indicate measurements at low salt (salt 1 and 2); green colors indicate measurements at high salt (salt 3 and 4; see Table 1 for salt conditions). (**G**) (Normalized) buckling points as a function of force. We averaged salt 1 and 2 to low salt (blue) and salt 3 and 4 to high salt (green). (**H**) Post buckling slopes, determined from the averaged extension-rotation data against force. Same color code as in G. (**I**) Buckling torque derived from the torque versus turn data. Color code as in G. Indicated with a filled, square symbol is the measured transition from B-DNA to P-DNA at 6.5 pN. Co-plotted in panels G–I is the Marko model (dashed lines) for dsDNA with fixed parameters: *C* (110 nm), *A* (43 nm for high salt conditions and 48 nm for low salt conditions) and *P*_lowsalt_ = 20 nm or *P*_highsalt_ = 15 nm.

Torque measurements on dsDNA were performed for a range of forces and salt conditions (see ‘Results’ section and Figures 2 and 3). In order to improve the signal-to-noise ratio in the torque and extension versus turns measurements, data for several independent DNA tethers were averaged for each condition. First, the responses of multiple DNA molecules to an applied number of turns were collected and analyzed; subsequently, the curves were aligned to yield single, averaged extension-rotation and torque-rotation curves for each force and salt condition. For measurements at forces of <1 pN, the rotation-extension curves are symmetric about zero turns (Figure 2A). For alignment, a Gaussian was fitted to the individual extension-rotation curves of independent DNA molecules (Figure 2C). The position of the maximum determined from the fit was defined as *L_k0_*, the number of turns at which the molecule is torsionally relaxed. The extension-rotation and torque-rotation curves were shifted along the turns-axis by –*L_k0_* to center the curves before averaging. For the averaging of the extension curves at forces >1 pN, a line was fitted to the constant region at negative turn values (Figure 2C). The first experimental value that fell below this line within experimental error was determined as the buckling point. Each curve was shifted along the turns-axis to align with the mean value of the buckling point of all measured DNA molecules for a given force and salt condition. Similarly, variations in the absolute extension of the molecule at zero turns were adjusted by shifting the curves. Finally, all curves for a given force and salt condition were averaged. For forces <1 pN, the data where binned in bins of two turns prior to computing the standard deviation (STD) and standard error of the mean (SEM) for both torque and extension as a function of applied turns (Supplementary Figure S13). For forces >1 pN, data were directly averaged (Supplementary Figure S14). Data points for each force and salt condition consist of at least 10 (and up to 45) independently measured molecules from at least two independent measurement runs, corresponding to an overall number of 475 measured molecules in this work. We note that the molecule-to-molecule variability (as measured by their standard deviation) of torsional measurements, in particular of the torsional stiffness, is about 10–20% in our current protocol, comparable with other single-molecule torque measurements (19,20,50). This means that averaging over multiple (≥10) independent molecules is required to reduce the standard error of the mean sufficiently to be able to probe or detect small changes. In the current study, we have systematically varied salt concentration and applied force for a total of 28 conditions; carrying out ≥10 measurements for this number of different conditions would have been impractical in a ‘one-molecule-at-a-time’ regime, highlighting the need for multiplexing.

## RESULTS AND DISCUSSION

### Principle of single-molecule torque measurements in magnetic torque tweezers

In MT, molecules of interest are tethered between a flow cell surface and micrometer-sized magnetic beads or particles (Figure 1A). External magnets are used to apply both forces and to control the rotation of the beads, therefore enabling the application of precisely calibrated stretching forces and control of the twist of the tethered molecules (52,69,70). Magnetic torque tweezers enable the measurement of torque at the single-molecule level by employing a magnet configuration with a weak angular trap (Figure 1B–D; see also below) and a tracking protocol that permits to track the rotation of the molecule around the tether axis (18,20,63,65). The basic principle of the torque measurement is as follows: tracking the rotation angle *Θ* initially for a torsionally relaxed molecule, the mean of the bead's rotation angle *Θ*_0_ indicates the equilibrium position of the rotational trap and the magnitude of the fluctuations around the equilibrium position allows us to calibrate the stiffness of the angular trap (*k_ROT_*):

(1) \begin{equation*}{k_{ROT}} = {k_B}T/Var(\Theta ) = {k_B}T/{(Std(\Theta ))^2}\end{equation*}

where *k_B_* is the Boltzmann constant and *T* the absolute temperature. After over- or underwinding the molecule by *N* turns, the restoring torque exerted by the molecular tether (*Γ_mol_*) gives rise to a shift in the mean angle position to a new angular position *Θ_N_* displaced from the initial equilibrium angle *Θ_0_*. The molecular torque can then be simply determined as the shift in mean angle multiplied by the rotational trap stiffness (Figure 1E):

(2) \begin{equation*}{\Gamma _{mol}} = - {k_{ROT}} < {\Theta _N} - {\Theta _0} >\end{equation*}

### Multiplexed magnetic torque tweezers

We have optimized our magnetic torque tweezers protocols to enable multiplexed measurements, i.e. to perform multiple single-molecule torque measurements in parallel. For parallel measurements, it is desirable to employ molecular constructs that avoid the need for multi-component assembly, such as the addition of a rotor bead to the DNA or a fiducial marker bead or rod to the main magnetic bead, to maximize the number of well-tethered and complete molecular constructs in a single field of view (FOV). In our mMTT scheme (Figure 1A), we therefore use simple double-stranded DNA constructs, attached with one end to the flow cell surface via multiple digoxigenin labels and to commercially available magnetic beads via multiple biotin-streptavidin linkages (see ‘Materials and Methods’ section), identical to constructs used routinely in conventional MT. We then exploit the particular geometry of bead fluctuations under a cylindrical magnet with a predominantly vertical field to track the beads’ rotation angles via their (*X,Y*)-positions.

#### Tracking rotation angle from (X,Y)-position

If the magnetic field is aligned exactly vertically (the so-called freely-orbiting MT or FOMT configuration with a single cylindrical magnet (60); Figure 1B), the bead is free to rotate about the tether axis and its thermal fluctuations trace out a doughnut shape (Figure 1B) in the (*X,Y*)-plane (60). Importantly, this configuration allows tracking of the rotation angle by conversion of the (*X,Y*)-position on the doughnut to polar coordinates (60). While the FOMT configuration enables observation of the unconstrained, rotational motion of the bead, it does not allow inducing twist. By adding a small magnet (or electromagnet (65)) to the cylindrical main magnet (Figure 1C), the magnetic field is slightly tilted (Figure 1D). The tilted field provides a (weak) rotational trap for the magnetic beads and their fluctuations in the (*X,Y*)-plane no longer trace out the full circle, but an arc-like shape (Figure 1C). The beads still experience an upward pulling force in *Z* and the weak rotational trap enables to twist the molecule of interest by rotating the magnet assembly (20,60). Importantly, the position in (*X,Y*) can still be used to track the rotation angle of the bead. In practice, it is not necessary to align the magnets in FOMT configuration to fit the doughnut pattern; instead, it is sufficient to record the (*X,Y*)-position while rotating the magnets and to fit a circle to the recorded trace in order to determine the radius of the molecules movement around its tether axis, which is in turn used to transform the recorded (*X,Y*)-position of the bead to polar coordinates (Supplementary Figures S1 and 2).

#### Optimization of the setup and trap stiffness for parallel torque measurements

There are conflicting requirements on the stiffness of the rotational trap in MTT, which in turn depends strongly on the magnetic field direction and strength. Equation (2) implies that for a given molecular torque, the shift in equilibrium angle is inversely proportional to the trap stiffness. As a consequence, if the trap stiffness is too high (≥1000 pN·nm/rad, corresponding to *Std*(*Θ*) <4°), typical torques exerted by DNA lead to very small shifts in the equilibrium angle (<1°) that are difficult or impossible to measure accurately (20). In contrast, if the trap stiffness is too low (≤30 pN·nm/rad or *Std*(*Θ*) >20°), the bead will not reliably follow the magnets’ rotation, therefore precluding systematic and controlled application of twist to the tether (Supplementary Figure S3). For multiplexed measurements, we have to ensure that the trap stiffness is within these limits for the entire field-of-view (400 × 300 μm^2^ at 40× magnification and 260 × 190 μm^2^ at 60× magnification in our setup). In addition, it is desirable to have a homogeneous gradient of the field along the *Z*-direction to ensure that all tethers experience similar stretching forces. To guide and optimize the choice of magnet configuration to satisfy these requirements, we carried out simulations of the axial and radial fields for our MTT magnet configuration.

We calculated the magnetic field vectors for the magnet configurations used in FOMT (Figure 1B) and (m)MTT (Figure 1C) with the equivalent source method and exploiting the superposition principle (Supplementary Figure S4) (57,65). The cylindrical magnet with a hollow center used in FOMT is equivalent to two superimposed solid cylindrical magnets with opposite magnetization and the effect of the side magnet in the (m)MTT is added by considering an additional, laterally offset cylindrical magnet. We calibrated the remanent field of our magnets through field measurements using a Hall Probe (Supplementary Figure S5). Furthermore we tested our field calculations against Hall probe field measurements along *Z* and *X* and found excellent agreement (Supplementary Figures S6 and 7). The magnetic field configurations for both FOMT and MTT (Figure 1D and Supplementary Figure S8) have the field and field gradient predominantly in the *Z*-direction. Importantly, the gradient of the magnetic field, which essentially sets the force exerted on the beads, varies only by 0.5% over 400 μm in *X* (which corresponds to the largest extension of the FOV used in this work) for the mMTT configuration. The essentially constant field gradient implies that all tethered beads in the FOV experience the same force, only limited by the ∼10% variation in total magnetization for the beads employed in this study (57,60,71).

For the FOMT configuration, the radial field (i.e. *X* or *Y*) is zero directly under the cylindrical magnet, while adding the side magnet in the (m)MTT configuration adds a field component in *X* (Figure 1D and Supplementary Figure S8). We found that for high-resolution torque measurement, in particular with the larger M270 beads, it is advantageous to lower the *X*-component of the field and, therefore, the trap stiffness. A displacement by ∼400 μm reduces the trap stiffness ∼3–4-fold, to 100–300 pN·nm/rad, compared to the alignment centered on the cylindrical magnet (Supplementary Figure S3). The rotational trap stiffness decreases with increasing distance of the magnet from the flow cell and, therefore, with decreasing force (Supplementary Figure S9). While the trap stiffnesses fall into the usable range of 30–1000 pN·nm/rad for all studied forces, we found it advantageous for low force torque measurements to bring the magnets closer to the flow cell (to 3–4 mm), corresponding to forces of 3–6 pN, for the segments of the torque measurement traces during which the magnets are actively rotated, to ensure that the beads reliably follow the magnets’ rotation.

For multiplexed torque measurements, it is important to not only have uniform forces across the FOV, but also uniform rotational trap stiffnesses. We found the variation in rotational trap stiffness for the same bead to only vary by <10% at different positions across the FOV (Supplementary Figure S10). The variability across the FOV is much less than the ≥20% bead-to-bead variability of the rotational trap stiffness at the same position in the flow cell (Supplementary Figure S10) that appears to stem from variations in the magnetic anisotropy of the beads employed (70,72). In practice, the variability in rotational trap stiffness is not a critical limitation, though, since the values tend to fall into the workable range of trap stiffnesses and since the same angle traces recorded for torque determination (Equation 2) are also used for the trap stiffness calibration via Equation (1). In practice, after discarding beads that are attached via multiple DNA tethers, nicked molecules or which are too close to other beads (which leads to tracking errors), we can simultaneously track currently up to 40 M270 beads or 80 MyOne DNA-tethered beads.

### Probing the torque response of dsDNA using mMTT

Our mMTT setup enabled us to systematically investigate the response of dsDNA under different salt conditions. We recorded the extension and the rotation angle around the tether axis, which is converted to torque via Equation (2), while systematically over- and underwinding DNA tethers (Figure 2). The extension versus rotation behavior of dsDNA depends strongly on the stretching force exerted on the molecules. For forces <1 pN, the extension-rotation curves for dsDNA are symmetric around zero turns (Figure 2A). Zero turns correspond to the torsional relaxed molecule. When the molecule is overwound (corresponding to positive turns and torques) or underwound (corresponding to negative turns and torques, respectively) at low forces (Figure 2A) its extension stays initially approximately constant. After a certain number of turns the buckling point *n_B_* is reached and the extension of the molecule decreases linearly with each additional turn of the magnets beyond the buckling point. Simultaneously, starting at zero turns, the torque exerted by the molecule increases linearly with the number of turns up to the point where the buckling torque is reached and the molecule undergoes the buckling transition (Figure 2D). Beyond the buckling point, the torque remains approximately constant and additional turns create additional turns of the plectonemic supercoils. The extension versus rotation curves become asymmetric for forces >1 pN (Figure 2B) due to torque-induced melting that occurs at −10 pN·nm (15,20,73) (Figure 2E). Positive supercoiling occurs up to forces of ∼6 pN. For the highest measured force, 6.5 pN, no buckling occurs at all, instead the DNA molecules undergo a transition from B- to P-DNA at ∼35 pN·nm (10,67,74) (Figure 2E).

The effective torsional stiffness of DNA can be determined from the experimentally measured torque versus turns response, by fitting a slope to the linear region of the torque versus turn response (Figure 2F):

(3) \begin{eqnarray*}C_{eff} &=& {L_C}/(2\pi N{k_B}T) \cdot {\Gamma _{mol,N}}\nonumber \\ &=& {L_C}/(2\pi {k_B}T) \cdot ({\Gamma _{mol,N}}/N) \end{eqnarray*}

Here *L_C_* is the contour length of the molecule and *N* the applied number of turns, thus *C_eff_* is in units of length and can be interpreted as a twist persistence length, in direct analogy to the bending persistence length *A*.

#### Extension-rotation and torque-rotation responses of dsDNA in different salt concentrations

We systematically recorded the extension and torque response of multiple DNA molecules and averaged the results for each force and salt condition (see ‘Materials and Methods’ section) including a total of 475 DNA molecules. For the measurements we chose a set of salt conditions (Table 1) that included monovalent salt concentrations from low (20 mM; ‘salt 1’) to high concentrations (500 mM; ‘salt 3’), including a point at near physiological monovalent salt concentration (100 mM; ‘salt 2’). In addition, we included measurements with 10 mM MgCl_2_ added (‘salt 4’); Mg^2+^ is the physiologically most abundant divalent ion and 10 mM Mg^2+^ is a typical ‘high’ concentration, used e.g. to induce RNA folding in *in vitro* measurements (21). We note that these salt conditions cover the range typically used in *in vitro* studies of for e.g. RNA folding, nucleic acid–protein interactions or nucleic acid nanotechnology.

Averaged high-resolution extension-rotation and torque-rotation data from mMMT measurements resolve a salt dependence of dsDNA when applying turns (Figure 3). The response in extension (Figure 3A–C) and torque (Figure 3D–F) are similar for salt 1 and 2 and for salt 3 and 4. For further discussion we averaged the low salt conditions (salt 1 and 2, blue colors) and independently averaged the high salt conditions (salt 3 and 4, green colors).

A large difference is observed between salt 1 and the higher salt conditions at 0.9 pN: While the extension-rotation curves are symmetric for the high salt conditions at this force, the response for salt 1 is asymmetric, indicating DNA melting upon underwinding. The lower salt concentration leads to a relative destabilization of the DNA double helix and thus to an earlier onset of DNA melting, consistent with previous reports (67,75).

#### Buckling points

The buckling points correspond to the number of turns at which the DNA molecule starts to form plectonemes when being over- or underwound. Here we report the buckling points in units of the supercoiling density (Figure 3G), which is the change in linking number (i.e. the applied number of turns) normalized to the number of turns in the torsionally relaxed helix, 1 turn per 10.5 bp. The transition from an overwound (or underwound for forces smaller 1 pN) but straight molecule (pre-buckling) to a buckled molecule depends on the ionic surrounding. For high salt concentrations (with less electrostatic repulsion) buckling occurs for a (slightly) lower number of applied turns, compared to low salt conditions (with stronger ionic repulsion) (41,42,45). The observed buckling points as a function of force are in reasonable agreement with a simple mechanical model of DNA due to Marko (76) (Figure 3G), as discussed below.

#### Post-buckling extension-rotation slopes

As negatively charged segments of DNA are brought into proximity in plectonemes, the radius (the size) of the plectonemes is strongly dependent on electrostatic interactions (41,45). For low salt conditions overall ionic screening is small, therefore electrostatic repulsion in the plectoneme is larger compared to the higher salt buffers, giving rise to a larger plectoneme radius and larger magnitude of the post-buckling slope at lower salt (Figure 3H), in agreement with previous measurements (19). The force-dependence of the post-buckling slope follows an empirical power law behavior, in quantitative agreement with the results of Mosconi *et al*. (19) (Supplementary Figure S16A). In addition, the force and salt dependence of the slope in the plectonemic region are again reasonably well described by the mechanical model for DNA (Figure 3H), as discussed in the next subsection.

#### Buckling torque

The measured molecular torque of dsDNA increases linearly up to the buckling transition for positive turns (41,45). We use the mean of the values in the plateau in the post-buckling regime to compute the buckling torque. Buckling torque increases with increasing force (see also Figure 2). The observed values are slightly lower for high salt compared to low salt (Figure 3I). Due to electrostatic repulsion at low ionic strength, dsDNA is able to store more molecular torque compared to high salt before undergoing the buckling transition. We observe the force-dependence of the buckling torques to follow the empirical power law proposed by Mosconi *et al*. (19) (Supplementary Figure S16B).

The observed buckling torques are in good agreement with the mechanical model by Marko (76) (Figure 3I). To fit the buckling points, slopes in the plectonemic regime and buckling torques in the framework of the Marko model, we used a fixed value for the intrinsic torsional stiffness *C* = 110 nm and the known values *A* = 48 and 43 nm for the bending persistence length at low and high salt, respectively. Our choice of *C*, notably taken to be independent of salt concentration, is a consensus value from previous studies (15,20,77) for the intrinsic torsional stiffness of DNA, corresponding to the high-force limit (see below). The values for the bending stiffness *A* under low and high salt conditions are typical average values for the corresponding salt conditions obtained in a number of different single-molecule studies (12,25–27,78) (see Supplementary Figure S1k of Ref. (27) for an overview). The stiffness of the plectonemic state *P* at low salt and high salt was first fitted individually to the buckling points, slopes in the plectonemic regime and the buckling torque data. We then averaged the resulting values for *P* and found *P* = 20 ± 4 nm and *P* = 15 ± 4 nm for low salt and high salt, respectively. We note that our value of 20 ± 4 nm at low salt is in agreement with the range of 21–27 nm suggested for these salt conditions; in addition, the observed decrease of *P* at high salt is as predicted by Marko (76).

In summary, our data on the behavior of DNA post-buckling is in agreement with previous results (19,41,45) and the effect of salt on DNA at the buckling point and in the post-buckling regime is at least semi-quantitatively described by the twistable worm-like chain model taking into account the known change in the bending persistence length and adjusting the torsional stiffness of the plectonemic state *P*. We find *P* to be 30% larger under our low salt conditions compared to high salt, which likely reflects the fact that in the plectonemes the DNA is sharply bent and segments of DNA are brought into close proximity.

#### The twist persistence length C_eff_

**Figure 4.**
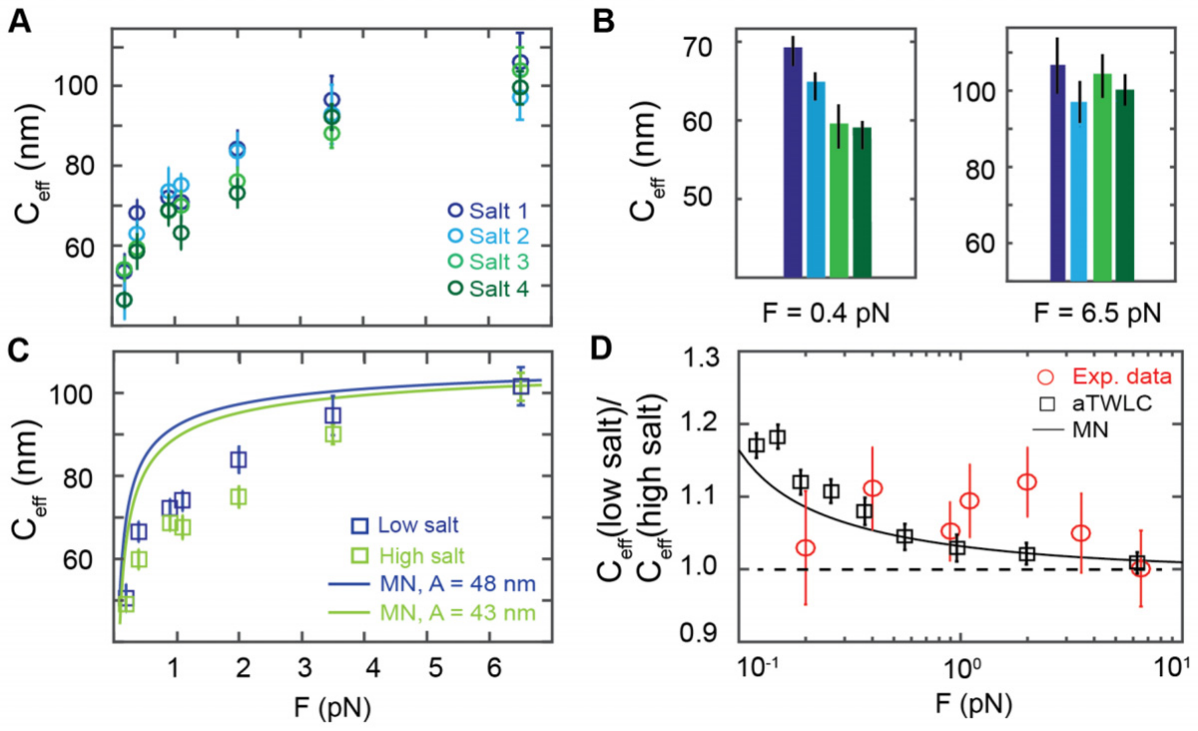
The effective twist persistence length of dsDNA for different salt conditions. The effective twist persistence length *C_eff_*as a function of force and salt. Data points are the means and standard errors of the mean from 10–45 independent molecules for each salt and force condition. (**A**) *C*_eff_ increases strongly with force, saturating for high forces at ∼100 nm for all salts. See Table 1 for salt conditions. (**B**) *C_eff_* for all four salt conditions at 0.4 pN and 6.5 pN. Color code as in A. (**C**) *low salt* (salt 1 and 2, blue) versus *high salt* (salt 3 and 4, green) torsional stiffness data (see main text) show a trend to be slightly lower for higher salt concentrations. The MN model is co-plotted (solid line), with fixed values *C* = 110 nm and *A* = 48 nm (low salt, blue) and *A* = 43 nm (high salt, green). (**D**) The ratio of the *low salt C_eff_* data and the *high salt C_eff_* as a function of force. Two models, the MN model (black line) and the aTWLC model (black squares) are co-plotted with the experimental data (red circles).

Next, we focus on the pre-buckling regime, which does not involve large, plectonemic deformations of DNA and is dominated by the effective torsional stiffness of DNA. Using our mMTT data, we determined the effective torsional stiffness *C_eff_* of DNA at varying forces and salt concentrations (Figure 4A), by fitting a slope to the torque versus applied turns data in the pre-buckling regime (Equation 3 and Figure 2F). The fitting region was kept constant for each force and it was carefully selected to only fit to the linear part of the curve, excluding data in the plectonemic and melting regimes.

The effective torsional stiffness increases with force for all salt conditions from ∼45 nm (at 0.2 pN) to ∼100 nm (at 6.5 pN). The decrease in effective torsional stiffness with decreasing force is due to correlations between bending and twisting (77,79) (see below) and has been seen in a number of previous studies (19,20,50,60,63).

Overall, the *C_eff_* values appear similar for the different salt concentrations, irrespective of the valency of the ions used (Figure 4A). At the measured high forces (3.5 and 6.5 pN), the measured torsional stiffness values converge and at 6.5 pN *C_eff_* is identical, within experimental errors, for all salt concentrations (Figure 4A and B). However, for lower forces (*F* < 3.5 pN) small but systematic differences between the different salt concentrations are apparent: the lower salt concentrations tend to give rise to higher *C_eff_* values in the low force regime (Figure 4A and B). Running an n-way ANOVA on the *C_eff_* values with salt and force as factors, we find that *C_eff_* varies statistically significantly with force (*P* = 1.3·10^−54^) and salt (*P* = 0.046). For clarity, we again averaged the two low salt concentrations and the two high salt concentrations; the resulting averaged values show the trend even more clearly (Figure 4C): An n-way ANOVA with salt and force as factors finds *P* = 0.0069 for the dependence on salt and *P* = 6.6·10^−57^ for the dependence on force. We note that the same trends are observed when measuring individual molecules under the different conditions (see e.g. Supplementary Figure S12); however, given the measurement uncertainty of ∼10–20% standard deviation, we focused on the averaged values for each conditions to draw statistical significant conclusions.

The effective torsional stiffness is independent of salt concentration at high forces (F > 6 pN), but systematically and statistically significantly lower for high salt in particular in the intermediate force regime (0.9–2.0 pN) (Figure 4C). The fact that the effective torsional stiffness deviates from the ‘true’ intrinsic torsional stiffness value and decreases with decreasing force has been investigated before and can be (at least qualitatively) understood by taking into account the coupling between bending and twisting fluctuations, in particular using the perturbative model by Moroz and Nelson (MN). Taking into account the known dependence of the bending persistence length on salt (and assuming a salt-independent value for the intrinsic torsional stiffness), the MN model predicts relative differences between the high and low salt conditions that are in good agreement, within experimental errors, with our measurements (Figure 4D, solid black line; reduced χ^2^ = 1.0). In contrast, the data are less consistent with the ratio of *C_eff_* at low versus high salt being equal to one (Figure 4D, dashed horizontal line; reduced *χ*^2^ = 1.92).

Unfortunately, the MN model does not quantitatively account for the force dependence of *C_eff_* and overestimates *C_eff_* at intermediate and low forces (0.2–2 pN) (Figure 4C). This shortcoming of MN theory has been described and investigated in a number of recent publications. Extensions of the MN model have been proposed, to account for the systematic deviations between the MN model and experimental data (80). One particular extension of MN is to extend the underlying isotropic rod model to include an anisotropy, i.e. distinguish between bending deformations between the two orthogonal directions that are perpendicular to the helix axis. First introduced by Marko and Siggia (81), this anisotropic rod model (aTWLC) has recently been investigated by Nomidis *et al*. by extensive coarse grained computer simulations (Nomidis, S., Kriegel, F., Vanderlinden, W., *et al*.; in preparation); the results suggest that introducing a non-zero twist-bend coupling constant (which is zero by definition in the isotropic rod model) can quantitatively account for the previously published *C_eff_* versus *F* data. Unfortunately, our current implementation of the coarse grained model does not explicitly account for electrostatics. However, again taking into account only the changes in bending persistence length with salt and keeping the intrinsic torsional stiffness fixed, the aTWLC model predicts differences of the high and low salt values for *C_eff_* very similar to the MN model and consistent with experimental data (Figure 4D, black squares; reduced *χ*^2^ = 1.15).

## CONCLUSION

We have developed and optimized an mMTT setup that enables parallel measurements of torque at the single-molecule level. Our mMTT approach is based on exploiting the particular geometry of DNA-tethered magnetic beads under a cylindrical magnet with a small side magnet, which allows us to track rotation angle from the beads’ motion in (*X,Y*). Using the mMTT implementation, we have obtained averaged extension-rotation and torque-rotation curves for DNA at varying salts and forces to, in particular, probe the effect of ionic conditions on the torsional stiffness of DNA. While we find the effective torsional stiffness to be ∼5–10% smaller for higher salt concentrations for forces <3 pN, *C_eff_* converges at high forces and there are no significant differences between salt conditions at 6.5 pN. The differences in the effective torsional stiffness at lower forces can be rationalized by mechanical models that only take into account the known change in the bending persistence length with salt and that assume a constant value for the intrinsic torsional stiffness of DNA. In summary, our results strongly suggest that while many properties of DNA under torsional strain, in particular of plectonemes, are salt-dependent, the intrinsic, high-force torsional stiffness of DNA is independent of ionic strength. Our results provide fundamental input for models and simulations of DNA under torsional constraint and will be valuable for understanding DNA in more complex biological contexts.

Our multiplexed torque protocol opens up the possibility to probe small changes in nucleic acid mechanics, through averaging, which would be difficult to detect in ‘one-molecule-at-a-time’ measurements. Here, we have focused on the salt-dependence, but our method would be equally applicable to study e.g. anisotropic effects in bending and twisting or the temperature dependence of elastic properties. Going beyond bare DNA, multiplexed torque measurements will also be applicable to other molecules (e.g. double-stranded RNA (27)) and enable us to probe DNA–drug and DNA–protein interactions.

## Supplementary Material

Supplementary Data
