# Supplementary material for: Probing the salt dependence of the torsional stiffness of DNA by multiplexed magnetic torque tweezers
Source: Nucleic Acids Res. 2017 Apr 29;45(10):5920–9. doi: 10.1093/nar/gkx280 (PMC5449586; doi:10.1093/nar/gkx280)
Supplement: Supplementary Data [file gkx280_Supp.pdf]

Probing the Salt Dependence of the Torsional Stiffness of DNA by Multiplexed Magnetic Torque Tweezers

Franziska Kriegel[1], Niklas Ermann[1,†], Ruaridh Forbes[2,§], David Dulin[2,3], Nynke H. Dekker[2] and Jan Lipfert[1,*]

[1]Department of Physics, Nanosystems Initiative Munich, and Center for Nanoscience, LMU Munich, Amalienstr. 54, 80799 Munich, Germany

[2]Department of Bionanoscience, Kavli Institute of Nanoscience, Delft University of Technology, Van der Maasweg 9, 2629 HZ Delft, The Netherlands

[3]Junior Research Group 2, Interdisciplinary Center for Clinical Research, Friedrich-Alexander-University Erlangen-Nürnberg (FAU), Hartmannstr. 14, 91052 Erlangen, Germany

[†]Current address: Cavendish Laboratory, University of Cambridge, Cambridge CB3 0HE, UK

[§]Current address: Department of Physics and Astronomy, University College London, Gower Street, London, WC1E 6BT, UK

[*]To whom correspondence should be addressed.
Phone: +49-89-2180-2005; Email: Jan.Lipfert@lmu.de

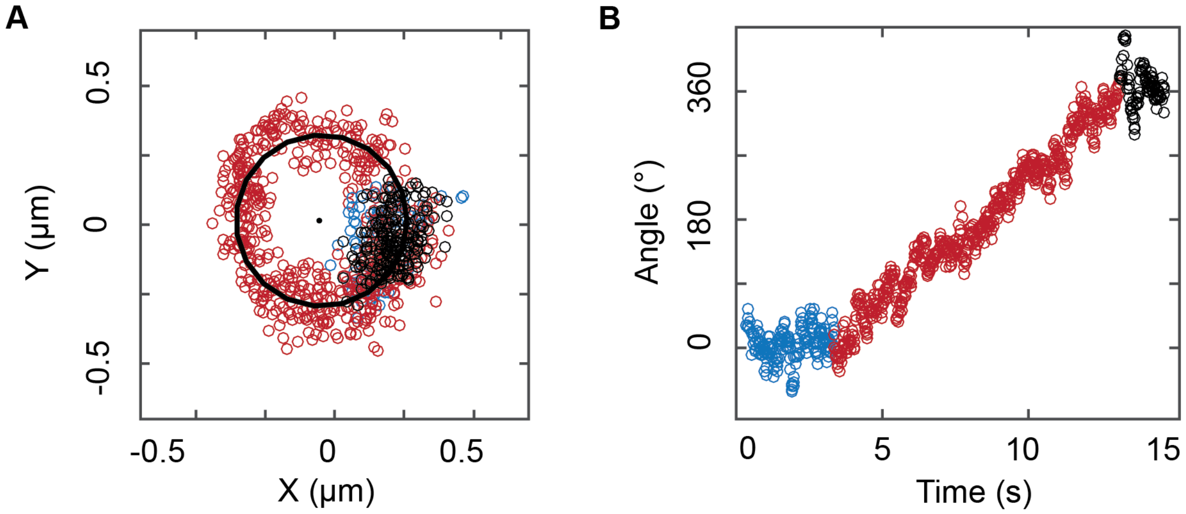

Supplementary Figure S1. Tracking of the bead's (*X,Y*) and angular position while rotating the magnets by one turn. (A) The bead's motion in the (*X,Y*)-plane before, after, and while rotating the magnets by one turn. The position of the bead before and after twisting the bead for one turn is shown in blue and black, respectively. The bead's position while turning the magnet -and thus the bead- is shown in red. A circle is fitted to the recorded data in order to determine the radius of the bead's motion and transform (*X,Y*)-positions to polar coordinates. (B) The trace of the rotation angle of the bead, obtained by coordinate transformation of the data in panel A. Same color code as in A. The measurement was performed with a MyOne bead at $F = 0.9$ pN in PBS buffer.

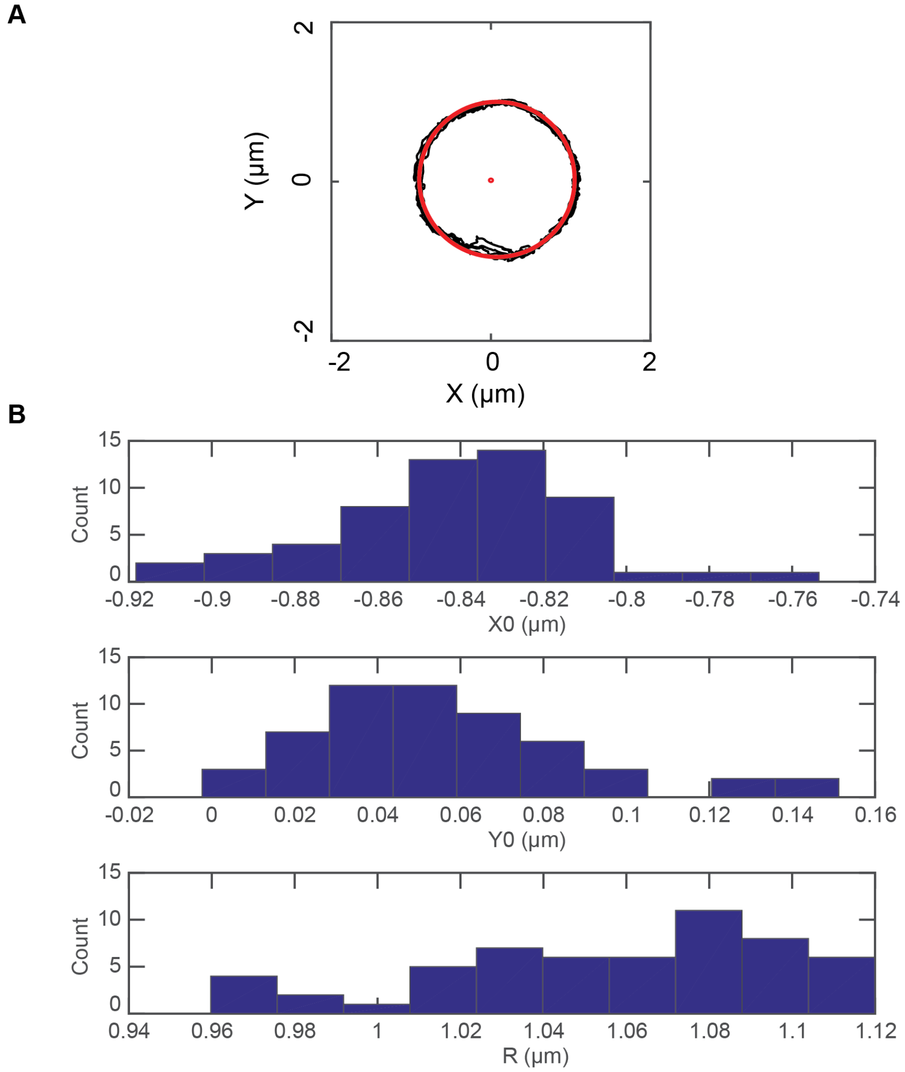

Supplementary Figure S2. Analysis of the (*X,Y*)-fluctuation and tether geometry during magnetic rotation in systematic torque measurements. To measure the torque and extension response of DNA tethers, we systematically over- and underwind them by applying integer number of turns with the magnets (typically 2 or 5 turns, depending on stretching force). The traces of (*X,Y*)-positions while rotating the magnets can be used to determine the attachment geometry of the DNA-bead system, defined by the center position (*X0*, *Y0*) and the radius of the circle *R* of the (*X,Y*)-fluctuation pattern, which are required to convert the observed (*X,Y*)-fluctuations to polar coordinates. It is, therefore, not necessary to first align the FOMT configuration (Figure 1B) prior to carrying out torque measurements. (A) Tracked (*X,Y*)-positions of a magnetic bead (M270) recorded while turning the magnets (in total five turns) in the mMTT (black). The circle fit is shown in red. The data and the fit were shifted for illustration such that the circle is symmetric around zero by subtracting the fitted (*X0*, *Y0*). (B) Histograms (all in µm) of fitted radii *R* and center positions (*X0*, *Y0*) obtained for one tethered molecule during a complete over- and underwinding cycle at one force consisting of 55 individual magnet rotation traces similar to the one shown in panel A. The fitted values for *R* and (*X0*, *Y0*) vary only by ~10 nm from trace to trace, which corresponds to an uncertainty of ~1% in the radius. We use the median of *R*, *X0* and *Y0*, in further analysis of the data set, in particular to transform the recorded (*X,Y*)-positions to polar coordinates. For this data set, the median values are 1.1151 µm, -0.8697 µm and 0.0466 µm, for *R*, *X0* and *Y0*, respectively.

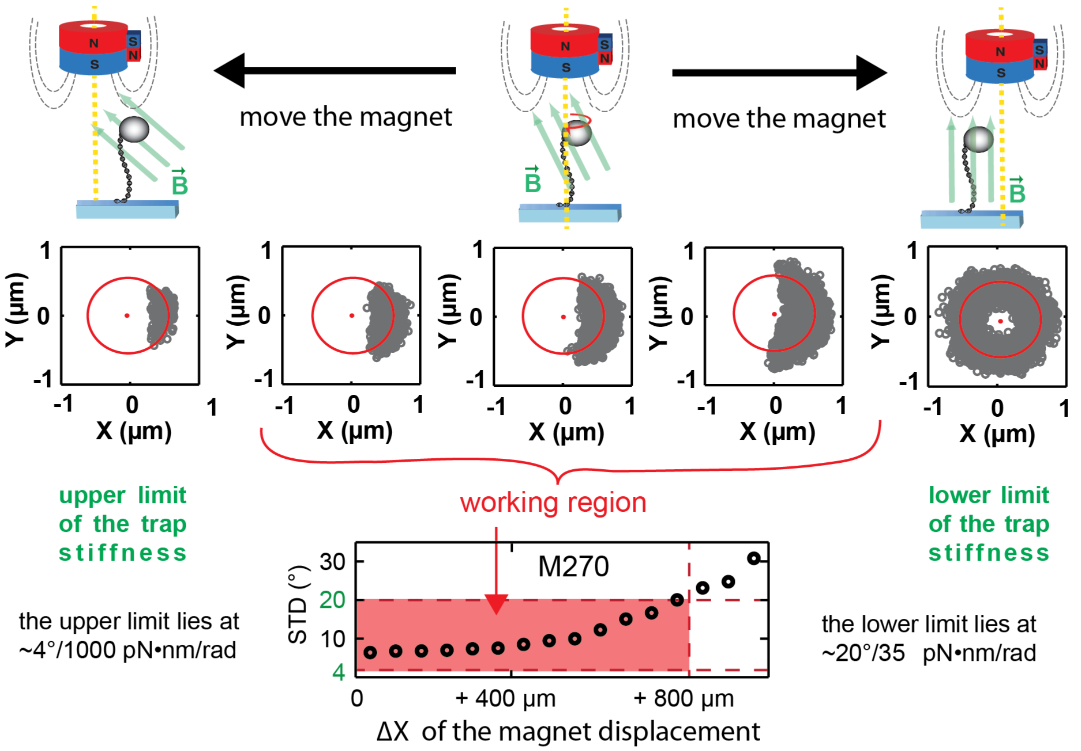

Supplementary Figure S3. Dependence of the torsional trap stiffness on magnet alignment. The stiffness of the angular trap depends strongly on the relative position of the bead to the magnet assembly. Top row: schematic of the magnet position relative to the DNA-tethered magnetic bead indicating how the displacement of the magnets affects the magnetic field at the position of the bead. Middle row: the bead's motion in the (*X,Y*)-plane for the relative bead-magnet alignment indicated above. Changes in the magnetic field direction when changing the magnet position lead to changes in the (*X,Y*)-fluctuations of the magnetic bead. Shifting the position of the magnet towards the direction of the side magnet leads to a shift of the magnetic field lines towards the vertical alignment. This in turn increases the extend of rotational fluctuations of the bead (corresponding to softer rotational trap stiffness). If the magnet assembly is shifted sufficiently far in the direction of the side magnet, the magnetic field at the bead's position becomes vertical and the trap stiffness is reduced to the point that thermal fluctuation are sufficient to trace at a full turn (rightmost column): the bead's motion in the (*X,Y*)-plane traces out a doughnut-like shape, as in the FOMT configuration. If the magnet is shifted in the opposite direction, the magnetic field lines are tilted towards the horizontal axis, such that the stiffness of the angular trap increases and the bead's motion is increasingly constrained.

Traces of bead fluctuation at different positions of the magnet were analyzed and the data converted to angular trap stiffness via Equation 1. Zero indicates the position where the magnetic field of the cylindrical magnet without side magnet is vertical, indicated by the fact that the bead's fluctuations trace a full circle in the (*X,Y*)-plane. When adding the small side magnet to the cylindrical magnet, the bead experiences a trap stiffness of ~200 pN·nm/rad (8°). It was previously shown that trap stiffnesses above ~1000 pN·nm/rad make it difficult to perform accurate measurements of typical molecular torques (1). To determine a lower limit for practical trap stiffnesses, we rotated the magnet assembly by one turn at each position and checked whether the bead follows the magnet rotation or not. The red box indicates the area in which the bead did follow the magnet rotation, beyond the red dashed line this was not the case. Below values of ~35 pN·nm/rad (or above standard deviation values of 20°), the stiffness of the trap is too soft to ensure bead rotations. The data shown are for a M270 bead measured at $F = 2$ pN in PBS buffer.

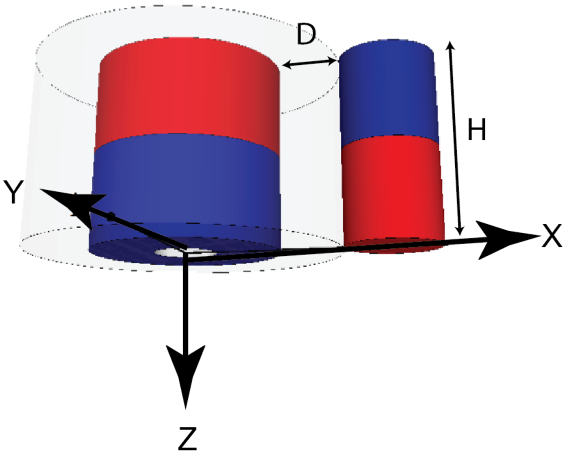

Supplementary Figure S4. Schematic of the magnet configuration in MTT and the principle of the 3D-field calculations. Between the cylindrical main magnet and the side magnet is a spacing of distance *D*. The center of the cylindrical magnet is chosen as center of the coordinate system. *Z* is pointing toward the flow cell and *X* is pointing towards the side magnet. The magnetic field is calculated using a semi-analytical description. The magnetic field is computed using the equivalent source method, essentially as described by Janssen *et al.* (2).

The field calculations for both the main cylindrical magnet and the side magnet are based on calculating the field of a cylindrical magnet. This can be achieved by solving the Biot-Savart law for an equivalent current around the curved surface of the cylinder. Since the radial field pointing outward from the central axis of the loop is symmetric about that axis, the field is divided into a radial (*X* and *Y* or *r*) and an axial (*Z*) component. The symmetric nature of the radial field means that this coordinate pair (*Z* and *r*), is sufficient to describe the full information in three dimensions: at an axial distance *Z* from the current loop and a radial distance *r* from the central axis, the field has a radial component $B_{loop,radial}$ pointing away from the central axis and an axis component $B_{loop,axial}$ in parallel with the central axis. The radial and axial fields from one current loop are given by

$$B_{\text{loop, radial}} = B_r \frac{z}{2\pi r\sqrt{z^2+(R+r)^2}}\left[E(m)\frac{R^2+z^2+r^2}{z^2+(R-r)^2} - K(m)\right]$$

$$B_{\text{loop, axial}} = B_r \frac{1}{2\pi\sqrt{z^2+(R+r)^2}}\left[E(m)\frac{R^2-z^2-r^2}{z^2+(R-r)^2} - K(m)\right]$$

$$\text{with } m = \frac{4Rr}{z^2+(R+r)^2}$$

where *Z* and *r* are the coordinates described above, B_R is the remanent field, *R* is the radius of the current loop and *K(m)* and *E(m)* are the complete elliptic integrals of the first and second kind.

For a coordinate system centered on the bottom central point of the cylindrical magnet with *Z* representing the direction of the central axis away from the magnet and *r* lying in the bottom plane (see above), the magnetic field is determined by integrating the field contributions from all loops:

$$B_{\substack{\text{radial}\\\text{axial}}}(z,r) = \int_z^{z+H} B_{\text{loop},\substack{\text{radial}\\\text{axial}}}(\hat{z},r)d\hat{z}$$

where the integration limits are given by the distance along Z over the height H of the magnet. Using this approach we first focus on the main magnet (i.e. the FOMT geometry), whose magnetic field in the (X,Y)-plane is described by (using the fact that a cylindrical magnet with a cylindrical aperture through the center can be modeled as two cylinders with different radii and opposite magnetizations):

$$B_{\binom{x}{y},\text{ main}}(x,y,z) = (-1)\begin{pmatrix} x \\ y \end{pmatrix}\frac{1}{\sqrt{x^2+y^2}}\Big[B_{\text{rad}}\Big(z,\sqrt{x^2+y^2},R_{\substack{\text{main}\\\text{outer}}},H_{\text{main}},B_{\text{r, main}}\Big) - B_{\text{rad}}\Big(z,\sqrt{x^2+y^2},R_{\substack{\text{main}\\\text{inner}}},H_{\text{main}},B_{\text{r, main}}\Big)\Big]$$

Where $R_{main,outer}$ is the outer and $R_{main,inner}$ is the inner radius of the cylindrical magnet, H_{main} is the height of the magnets and $B_{r,main}$ the magnetization of the magnet.

The field of the side magnet in (X,Y) is given by:

$$B_{\binom{x}{y},\text{ side}}(x,y,z) = \begin{pmatrix} (x-D) \\ y \end{pmatrix}\frac{1}{\sqrt{(x-D)^2+y^2}}B_{\text{rad}}\Big(z,\sqrt{(x-D)^2+y^2},R_{\text{side}},H_{\text{side}},B_{\text{r, side}}\Big)$$

With R_{side} being the radius of the side magnet, H_{side} being its height and $B_{r,side}$ its magnetization. By the superposition principle the total field's components along X and Y are given by:

$$B_{\binom{x}{y}}(x,y,z) = B_{\binom{x}{y},\text{main}}(x,y,z) + B_{\binom{x}{y},\text{side}}(x,y,z)$$

With the same argument, the total field's projection onto Z results from

$$B_z(x,y,z) = B_{\text{z, main}}(x,y,z) + B_{\text{z, side}}(x,y,z) =$$
$$(-1)\Big[B_{\text{ax}}\Big(z,\sqrt{x^2+y^2},R_{\substack{\text{main}\\\text{outer}}},H_{\text{main}},B_{\text{r, main}}\Big) - B_{\text{ax}}\Big(z,\sqrt{x^2+y^2},R_{\substack{\text{main}\\\text{inner}}},H_{\text{main}},B_{\text{r, main}}\Big)\Big]$$
$$+ B_{\text{ax}}\Big(z,\sqrt{(x-D)^2+y^2},R_{\text{side}},H_{\text{side}},B_{\text{r, side}}\Big)$$

which adds the last dimension to the overall field. The equations for $B_{x,y}$ and B_z give the complete description of the magnetic field in the MTT. The calculations were implemented in custom MATLAB routine, which are available from the authors upon request.

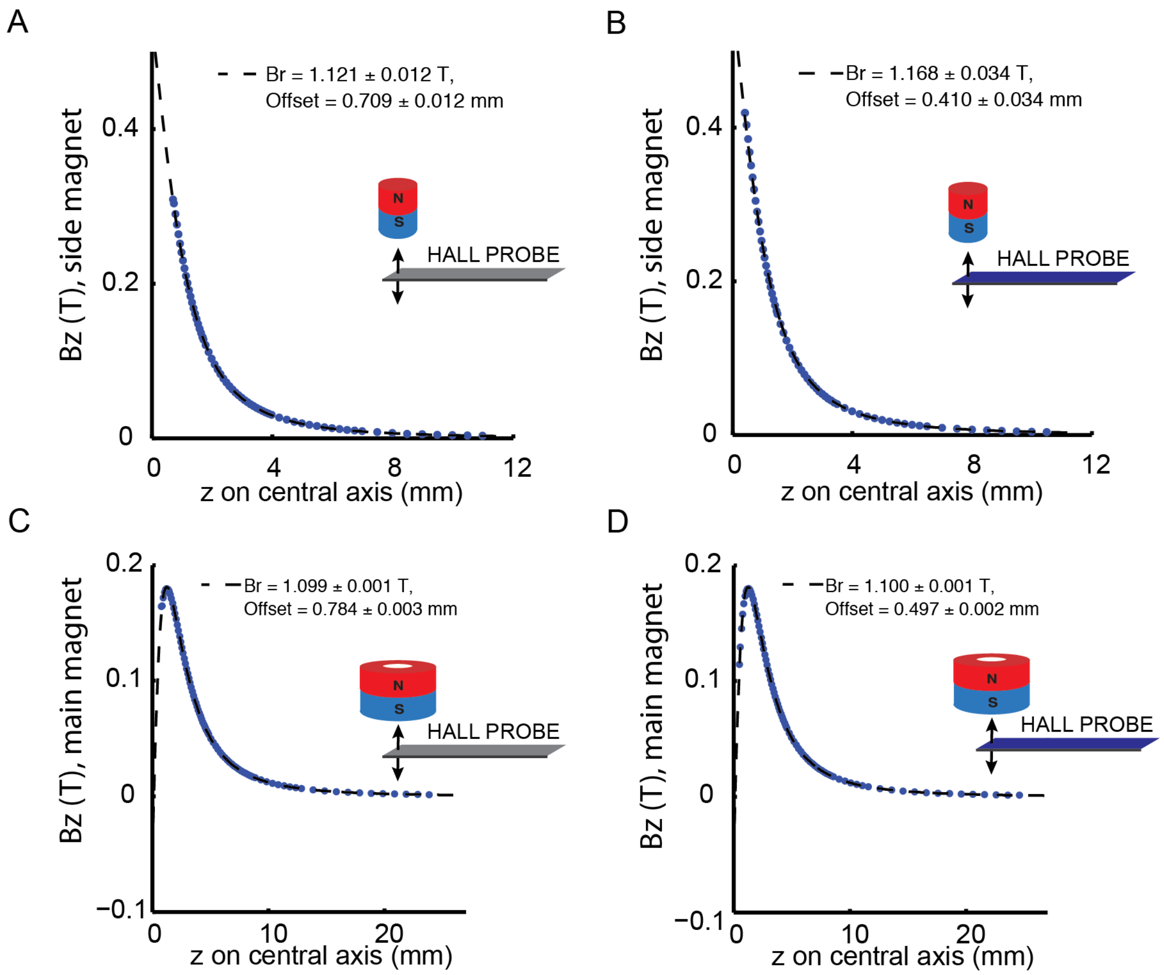

Supplementary Figure S5. Determination of the remanent magnetic field (*Br*) for the main magnet and the side magnet. To calibrate our field calculations, we carried out magnetic field measurement with a Hall probe (AS-NTM-2 with Teslameter FM 302, Projekt Elektronik, Berlin, Germany) to determine the remanent magnetic field of both the side magnet (panel A and B) and the main magnet (panels C and D). Since the exact position of the Hall sensor in the probe casing was not known, we carried out the Hall probe measurements with either of its two sides (indicated by two colors: grey and blue) facing the magnet. The exact distance of the Hall probe casing surface to the measurement element was treated as a fitting parameter named *offset*. The insets illustrate the direction of the Hall probe and how it was approached towards the magnet. Blue data points are measured values and the black dashed-line is a fit of the magnetic field (as described above) with *Br* and the *offset* as fitting parameters. The error of the Hall probe. i.e. of the measured field values, is small at 0.05 % (vendor's specifications) and, therefore no error bars are shown for the experimental data. By combining the two measurement directions, we found $B_{r,side\ magnet}$ = 1.14 T ± 0.09 T and $B_{r,main\ magnet}$ = 1.10 T ± 0.01 T. These experimentally determined *Br* values are used for all field calculations shown below.

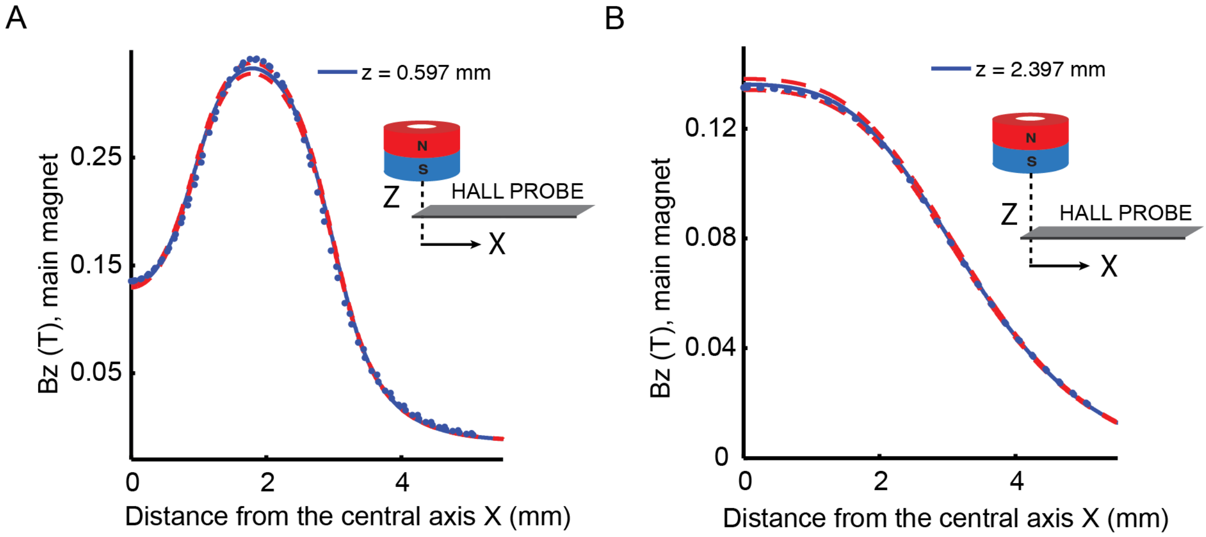

Supplementary Figure S6. Field calculations for *Bz* and comparison to experimental data. With the remanent field values calibrated (Supplementary Figure S5), we simulated the field in the axial (*Z*) direction, at a given (fixed) axial position as a function of the radial (or X in this case) position. For comparison, we again obtain Hall probe measurements of the field, as indicated schematically in the insets. Blue circles indicate experimentally measured values. The blue line shows the calculated axial field using the previously determined values for *Br*. The red dashed line presents the error on the simulation using the upper and lower deviation of the measured remanent field values. Overall, we find excellent quantitative agreement between the field calculations and the experimental data.

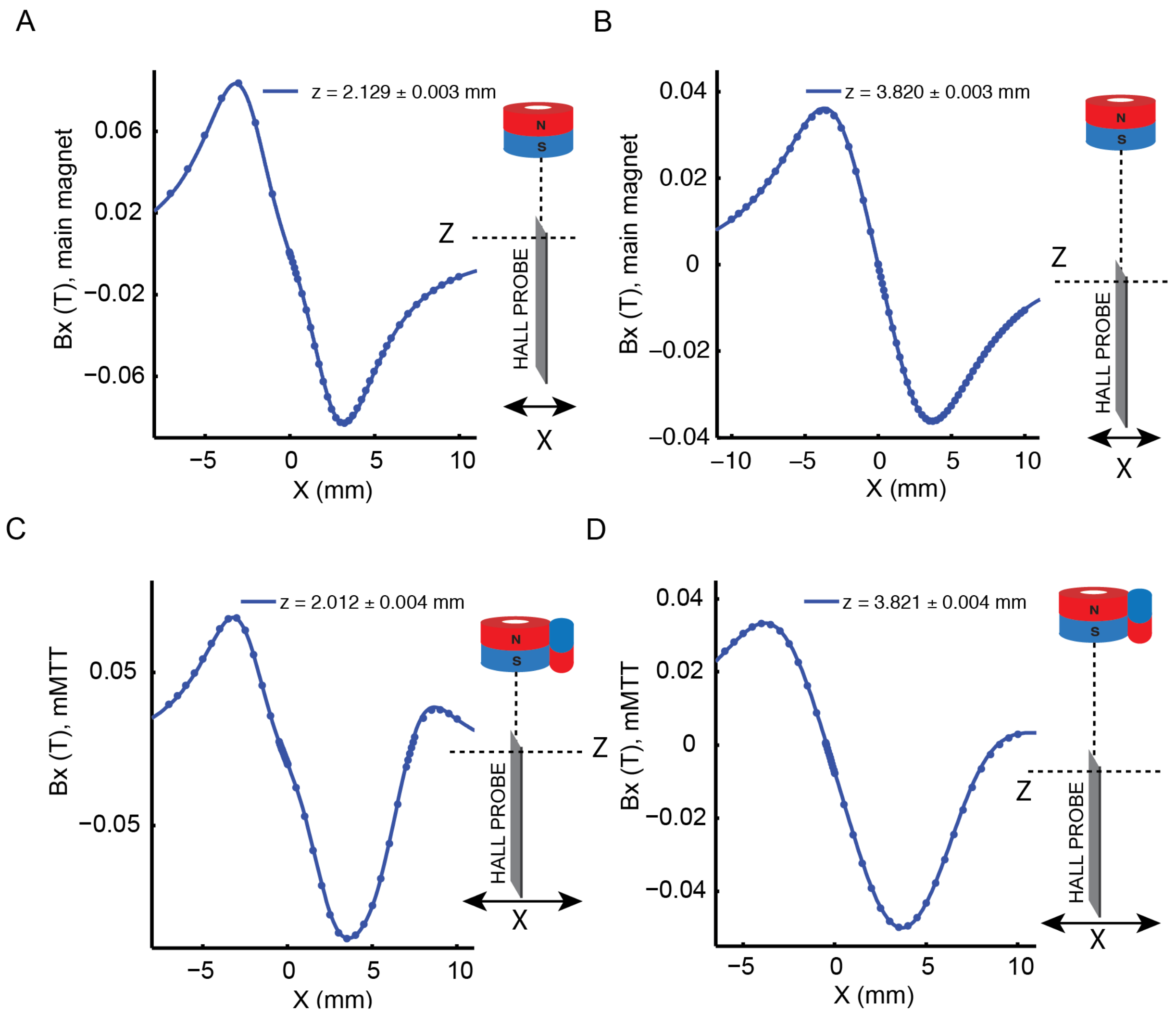

Supplementary Figure S7. Field calculations for *Bx* and comparison against experimental data. (A and B) For two fixed values of *Z* we measured the radial field component as a function of *X* (at $Y = 0$) and compare with the field calculations for the central hollow cylindrical magnets only (i.e. the FOMT magnet configuration) (A and B) and for the central magnet with added side magnet (i.e. the MTT magnet configuration) (C and D). The insets illustrate how we performed the Hall Probe measurements. Here, we move the Hall Probe along *X* at a fixed distance *Z*. Due to the uncertainty of the exact position of the Hall sensor along Z, the distance Z from the magnet to the sensor served as a fitting parameter with the respective results shown as insets. Experimental data is shown as blue circles, the simulation as solid blue lines. Error bars are omitted for clarity as they lie close to the blue line. Again, we find excellent agreement between our field calculations and the experimental data, confirming the validity of our field simulations.

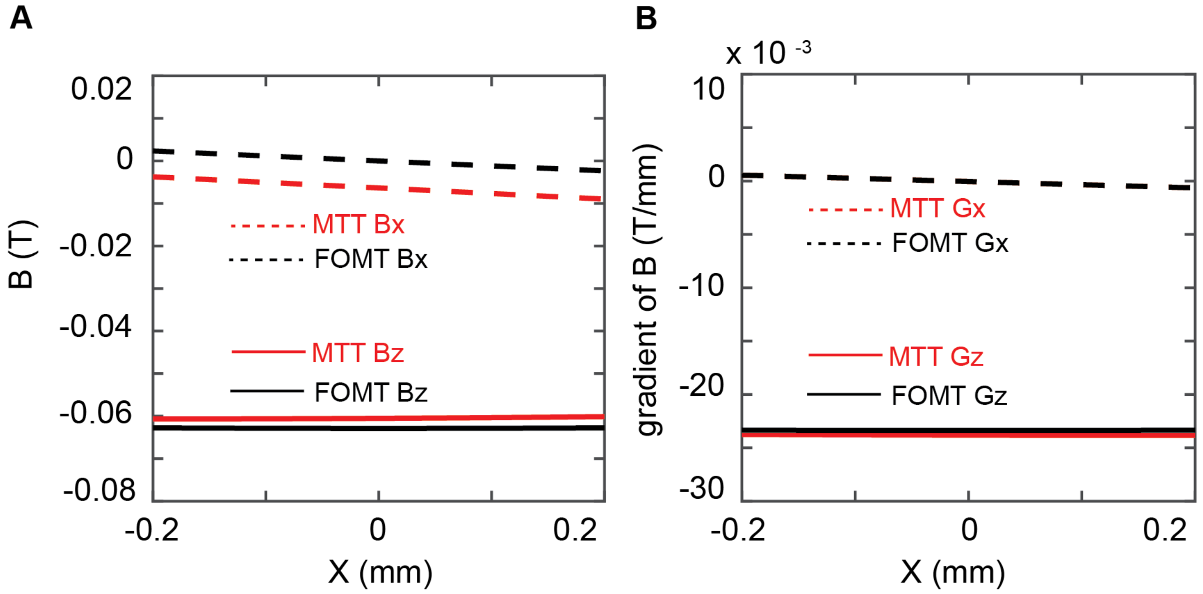

Supplementary Figure S8. Magnetic field calculations for FOMT and MTT magnet configurations. Magnetic fields and field gradients exerted by the FOMT and MTT magnet geometries, respectively, at a distance of 4.4 mm from the bottom face of the cylindrical magnet, which corresponds to a typically intermediate magnet position used on the DNA torque measurements. Calculations were performed with the experimentally determined magnetization values as described above (Supplementary Figures S4 and S5). $X = 0$ is defined as the position of unconstrained rotational motion in the FOMT setup (blue), i.e. where the bead's fluctuations trace out a full circle. The MTT setup results from adding a side magnet on the side of positive X. (A) Magnetic fields as a function of X-position. Solid lines indicate the (axial) field along Z and dashed lines indicate the (radial) in X-direction. It can be seen that adding the side magnet to the main magnet leads to a shift in Bx towards negative values. (B) Magnetic field gradients as a function of X-position in Z-direction (solid line) and along the X-direction (dashed line). The field gradients in X-direction are small and essentially identical for the MTT and FOMT magnet geometries. The field gradient is related to the applied force on the bead and thus to the stretching forces exerted on the molecule (3). The field gradient is predominantly in the Z-direction, corresponding to an applied stretching force along the Z-direction. The extent of the X-axes in panels A and B corresponds to the largest dimensions of the field of view (FOV) in our setup (400 µm). The change in the field gradient in the MTT configuration over the size of the FOV is ≤ 0.5 %; consequently, the applied stretching forces are very homogeneous across the FOV. The difference in field gradient between the FOMT and MTT configurations is ≤ 2 %. The upward stretching forces are, therefore, almost identical for the FOMT and MTT configurations. Similar conclusions can be drawn from calculations at different magnet heights (in the range used in our measurements) and if the FOV is shifted along X.

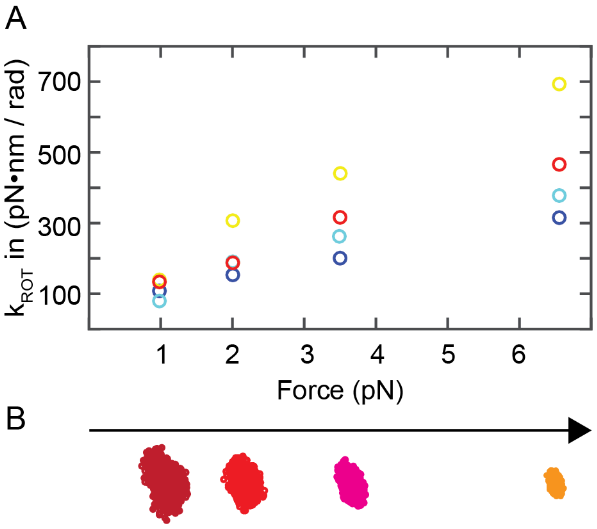

Supplementary Figure S9. The stiffness of the rotational trap increases with increasing force. (A) Measured trap stiffness for four M270 beads (different colors correspond to different beads) as a function of the applied stretching force. In order to change the force, the magnets are moved in *Z*-direction. The magnetic field and its gradient changes with the height of the magnets and thus both the force and trap stiffness changes with magnet height (1, 2). The closer the magnets, the higher the force and the stronger the angular trap. For this magnet alignment, the trap stiffness for four different beads at 6.5 pN varies from 300 to 700 pN·nm/rad, which still lies within the workable range to perform torque measurements. The spread of the measured rotational trap stiffnesses at a given magnet height (or force), is likely due to bead-to-bead variations of their magnetic anisotropy (4) (see also Supplementary Figure S10). (B) Bead fluctuations in the *(X,Y)*-plane for one particular bead held at the stretching forces indicated by the axis on top.

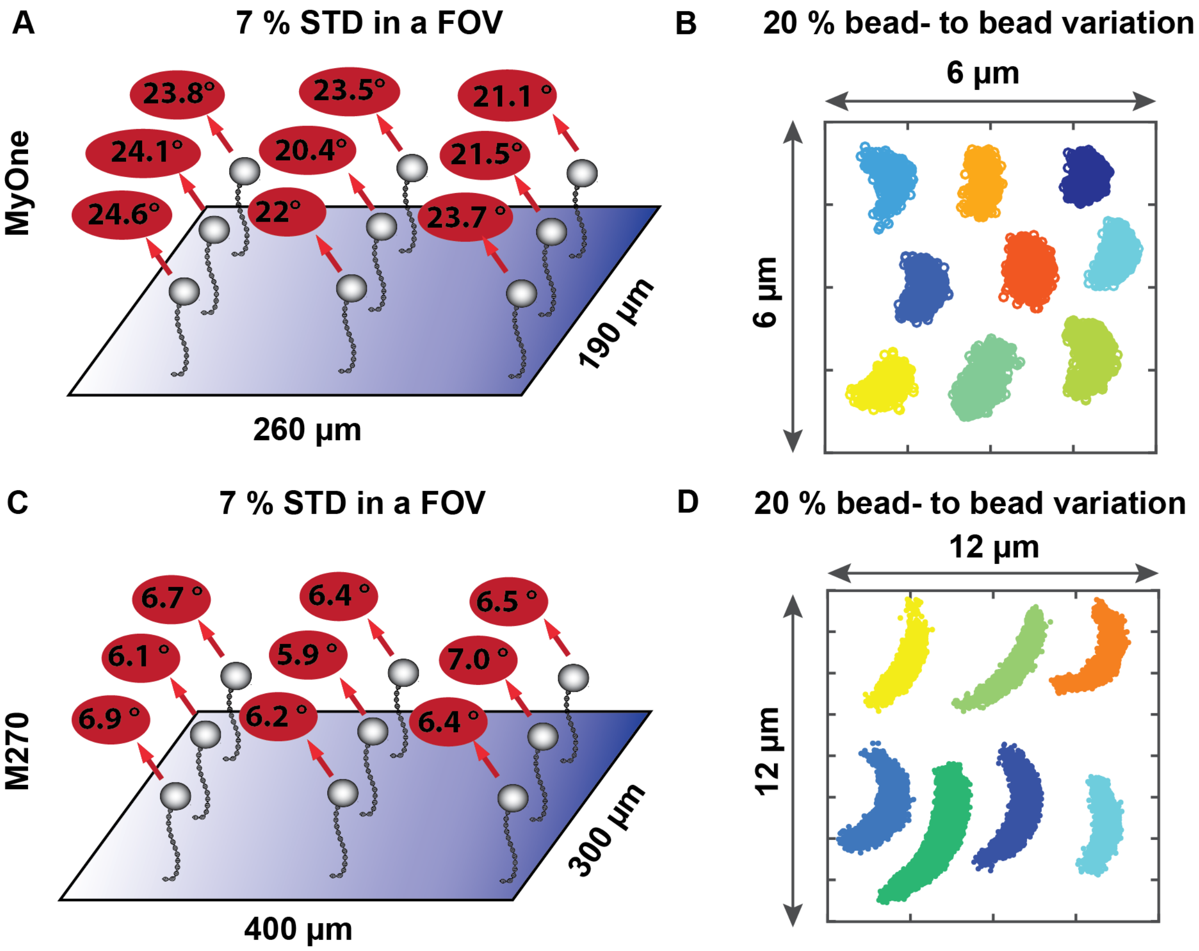

Supplementary Figure S10. Bead-to-bead variation and variation across the field of view of the rotational trap stiffness. Measurements for MyOne (A,B) and M270 (C,D) beads at a magnet height of 4 mm above the flow cell surface. (A, C) The position of one particular bead was varied across the FOV by moving the flow cell with respect to the camera and magnet assembly and at each position the standard deviation of the angular fluctuations was measured. The positions in the FOV are shown schematically and the corresponding standard deviations are annotated. The variation of the standard deviations of the angular fluctuations of a bead across the FOV is ~7 %. (B, D) In order to determine the bead-to-bead variation of the rotational trap stiffness we measured several beads in the same position under the magnets, within a fixed region of interest (6 x 6 µm for MyOne and 12 x 12 µm for M270 beads). This ensures that the relative position of the magnets to the bead center was only allowed to vary by a few µm. The different colors represent fluctuations of different beads and are placed in the area for illustration. Bead fluctuations within this figure are shown 6x larger than in reality. The bead-to-bead variation was found to be about 20 % (measurements were performed for 10 beads in PBS buffer).

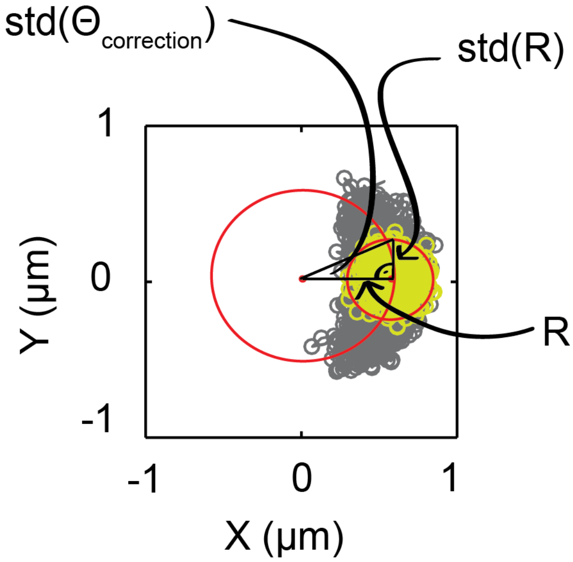

Supplementary Figure S11. Cross-talk correction of the angular fluctuations. Due to thermal noise the bead fluctuates in the *(X,Y)*-plane (shown in yellow) even in the absence of rotational fluctuations. The magnitude of these transverse fluctuations in the magnetic tweezers is given by the classic "inverted pendulum" argument and is determined by the applied force and tether length. In the MTT/FOMT configuration the radial fluctuations are representative of the transversal fluctuations and can be used to calibrate the stretching force (5). However, since the transverse fluctuations occur both in *X* and *Y*, they give rise to apparent fluctuations in the angle, even in the absence of bead rotation. To correct for this cross-talk, we compute the magnitude of the angle fluctuations due to the "standard magnetic tweezers" transverse fluctuations from the standard deviation of the radial fluctuations (std(*R)*) and the fitted radius (R):

$$\mathrm{Var}(\Theta_{correction}) = (\arctan(std(R)/R))^2$$

When calculating the stiffness of the angular trap (Equation 1), we employ the corrected variance of the angle fluctuations:

$$\mathrm{Var}(\Theta) = \mathrm{Var}(\Theta_{measured}) - \mathrm{Var}(\Theta_{correction})$$

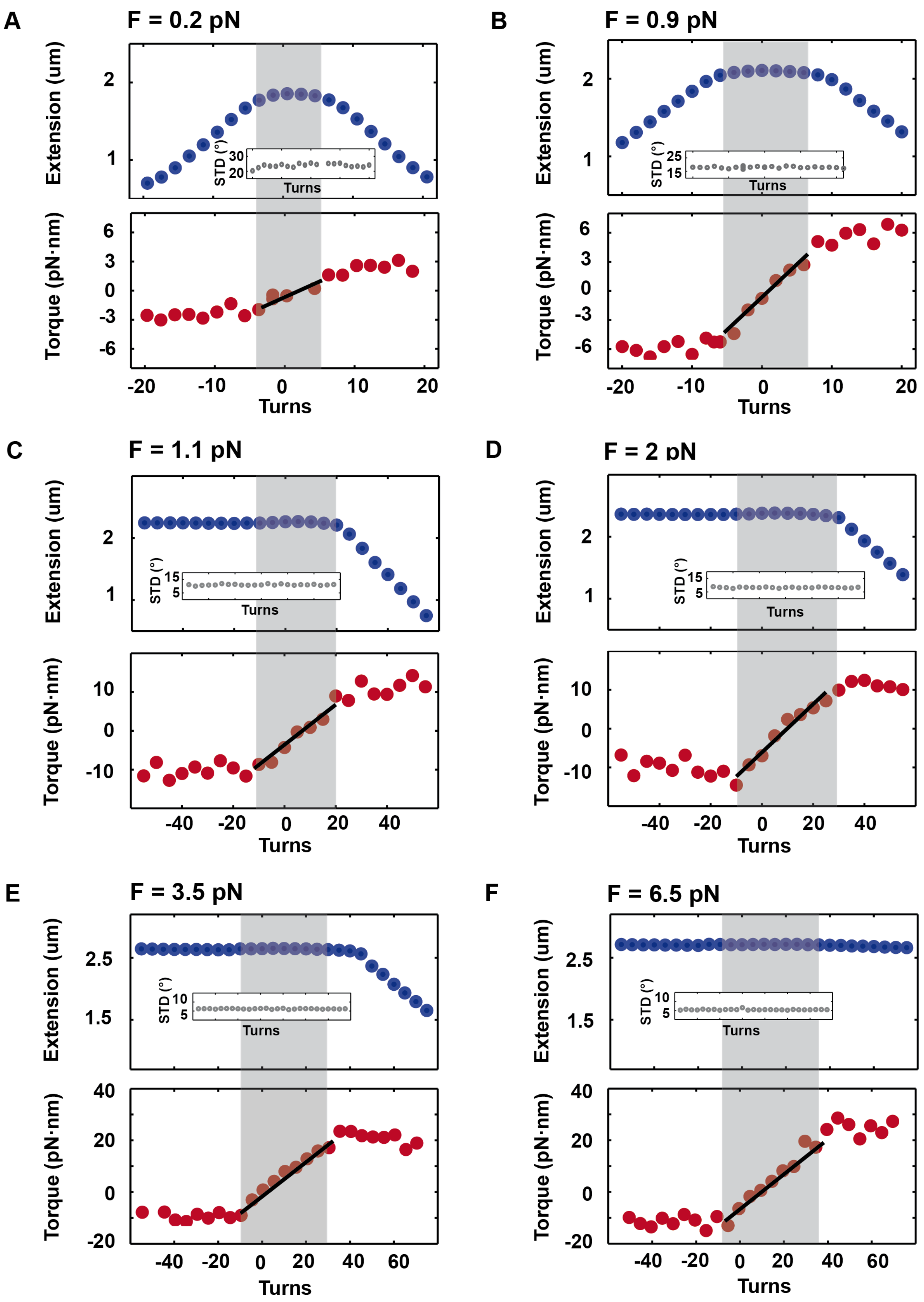

Supplementary Figure S12. Representative torque measurements of dsDNA for a single molecule at different forces. Panels A to F show torque measurements of dsDNA at 100 mM NaCl (in TE buffer, pH 7.4) at varying forces for one particular molecule. The insets show the standard deviation (which is related to k_{ROT} by Equation 1) recorded for each measurement point.

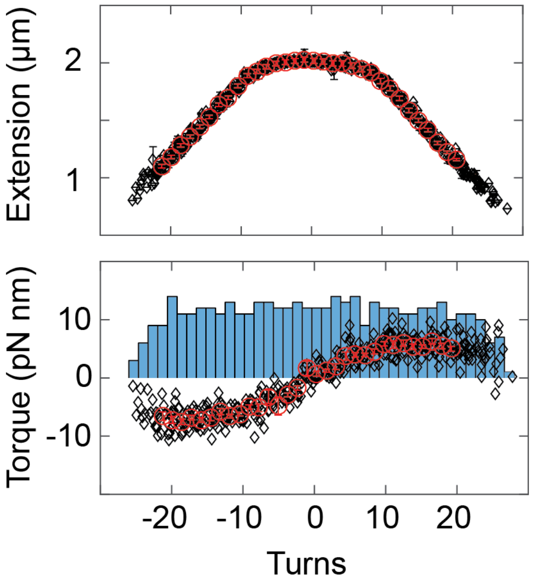

Supplementary Figure S13. Multiple bead measurements: Averaging algorithm for symmetric extension-rotation curves. Data shown were recorded at 0.4 pN (using MyOne beads) and in TE buffer supplemented with 100 mM NaCl. The extension-rotation data of each molecule were first analyzed individually by fitting a Gaussian to determine L_{k0} of the molecule. The extension as well as the torque data were shifted by $-L_{k0}$ on the turn axis in order to be symmetric around zero turns. The extension of the molecule was adjusted by correcting each individual extension according to the deviation to a defined reference curve. Corrected data for multiple molecules for the extension vs. turns and for torque vs. turns are shown as black diamonds. Next, the data were binned. The blue bars in the background of the torque-rotation data indicate the number of points in each bin. Values within each bin were averaged to yield the final averaged extension vs. turns and torque vs. turns data (red circles).

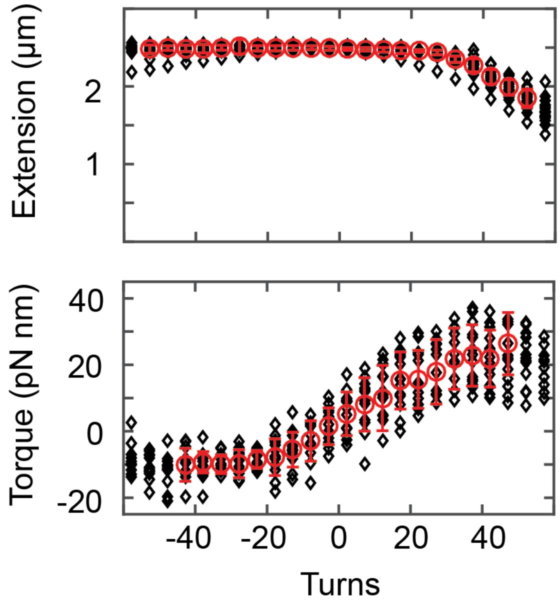

Supplementary Figure S14. Multiple bead measurements: Averaging algorithm for asymmetric extension-rotation curves. Data shown within this figure were recorded at 3.5 pN (M270 beads) and in TE buffer supplemented with 100 mM NaCl. The extension-rotation data of each molecule were first analyzed individually. In order to overlay the curves along the turns axis, the buckling point of each curve was determined by fitting a horizontal line to the extension-rotation data in the range where the extension is approximately constant. The first experimental value of the extension data (within error) at positive turns that falls below this line is defined as the buckling point (Figure 2C). Each curve is then shifted to the mean value of all collected buckling points at that particular force. The extension of the molecule was adjusted by correcting each individual extension according to the deviation to a defined reference curve. Torque values were shifted to -10 pN·nm for -20 turns (or smaller), assuming that torque-induced melting occurs for these values. All extension or torque values at the same number of turns are averaged, to obtain final averaged extension vs. applied turns and torque vs. applied turns curves.

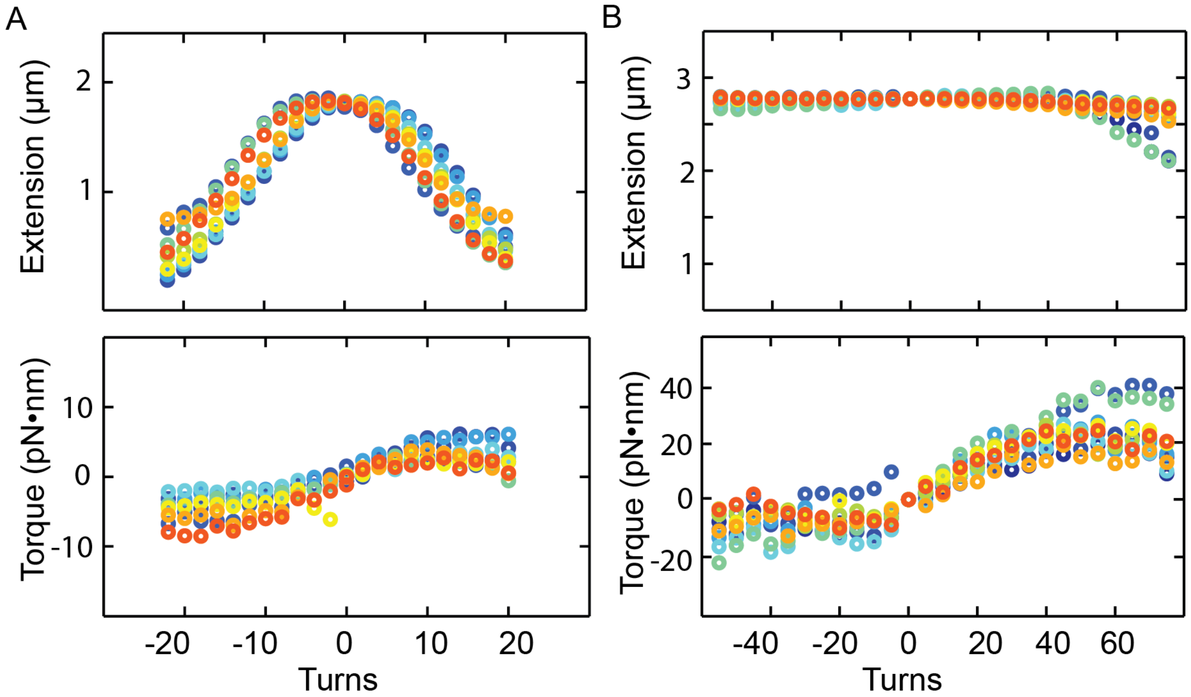

Supplementary Figure S15. Multiplexed extension and torque measurements. Each color corresponds to one particular molecule, all measured at the same time in one mMTT measurement run. No averaging or shifting is performed for these data sets. Shown here, are 8 tethers using MyOne beads at 0.4 pN (panel A) and 9 DNA tethers using M270 beads at 6.5 pN (panel B), each measured simultaneously. Measurements were performed in TE buffer with 100 mM NaCl and at pH 7.4.

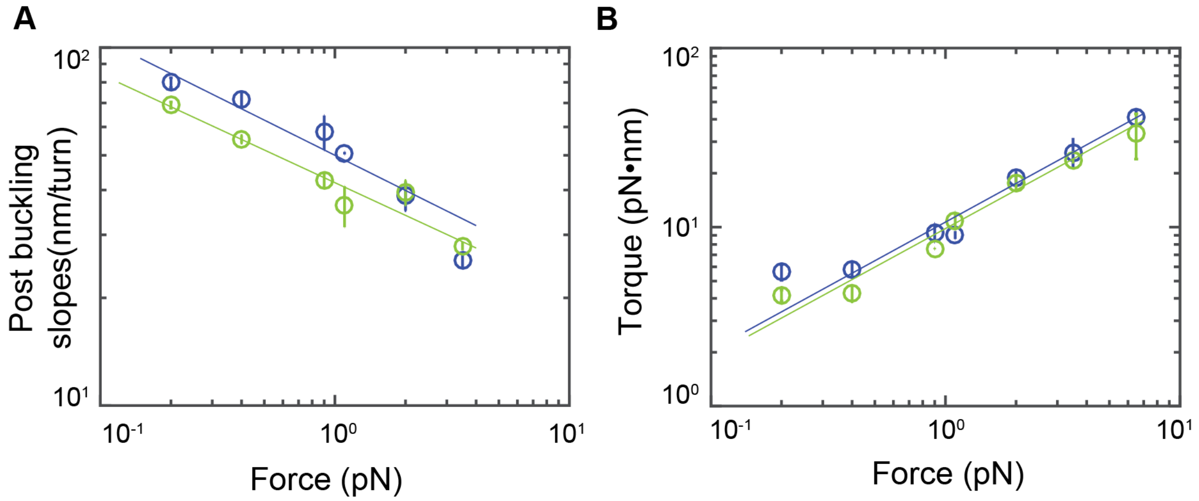

Supplementary Figure S16. Double-logarithmic plots of the measured post buckling slopes vs. force (A) and buckling torques vs. force (B) for low salt (blue) and high salt (green) data. These are the same data as Figure 3H and I. We find a power law dependence for the post buckling slopes with an exponent of −0.33 (95% confidence intervals: −0.19 to −0.46) at low salt and −0.30 (95% confidence intervals: −0.21 to −0.49) at high salt (solid lines in A). Similarly, for the buckling torques, we find exponents of 0.72 (95% confidence intervals: 0.48 to 0.95) at low salt and 0.71 (95% confidence intervals: 0.52 to 0.91) at high salt (solid line in B). The fitted exponents are in quantitative agreement with Mosconi *et al.* (6). Additionally, we see similar trends for the salt dependence. The exponential fit was performed between 0.2 and 3.5 pN.

Supplementary References

1. Lipfert,J., Kerssemakers,J.W.J., Jager,T. and Dekker,N.H. (2010) Magnetic torque tweezers: measuring torsional stiffness in DNA and RecA-DNA filaments. *Nature Methods*, **7**, 977–980.

2. Janssen,X.J.A., Lipfert,J., Jager,T., Daudey,R., Beekman,J. and Dekker,N.H. (2012) Electromagnetic torque tweezers: a versatile approach for measurement of single-molecule twist and torque. *Nano Lett.*, **12**, 3634–3639.

3. Lipfert,J., Hao,X. and Dekker,N.H. (2009) Quantitative modeling and optimization of magnetic tweezers. *Biophysical Journal*, **96**, 5040–5049.

4. van Oene,M.M., Dickinson,L.E., Pedaci,F., Köber,M., Dulin,D., Lipfert,J. and Dekker,N.H. (2015) Biological magnetometry: torque on superparamagnetic beads in magnetic fields. *Phys. Rev. Lett.*, **114**, 218301.

5. Lipfert,J., Wiggin,M., Kerssemakers,J.W.J., Pedaci,F. and Dekker,N.H. (2011) Freely orbiting magnetic tweezers to directly monitor changes in the twist of nucleic acids. *Nat Commun*, **2**, 439–9.

6. Mosconi,F., Allemand,J.F., Bensimon,D. and Croquette,V. (2009) Measurement of the torque on a single stretched and twisted DNA using magnetic tweezers. *Phys. Rev. Lett.*, **102**, 078301.
